# Wilms tumor 1 impairs apoptotic clearance of fibroblasts in distal fibrotic lung lesions

**DOI:** 10.1172/JCI188819

**Published:** 2025-06-10

**Authors:** Harshavardhana H. Ediga, Chanukya P. Vemulapalli, Vishwaraj Sontake, Pradeep K. Patel, Hikaru Miyazaki, Dimitry Popov, Martin B. Jensen, Anil G. Jegga, Steven K. Huang, Christoph Englert, Andreas Schedl, Nishant Gupta, Francis X. McCormack, Satish K. Madala

**Affiliations:** 1 Division of Pulmonary, Critical Care and Sleep Medicine, Department of Internal Medicine, University of Cincinnati, Cincinnati, Ohio, USA.; 2 Gordian Biotechnology, South San Francisco, California, USA.; 3 Division of Biomedical Informatics, Cincinnati Children’s Hospital Medical Center, Cincinnati, Ohio, USA.; 4 Department of Pediatrics, University of Cincinnati College of Medicine, Cincinnati, Ohio, USA.; 5 Division of Pulmonary and Critical Care Medicine, Department of Internal Medicine, University of Michigan Medical School, Ann Arbor, Michigan, USA.; 6 Molecular Genetics Lab, Leibniz Institute on Aging – Fritz Lipmann Institute, Jena, Germany.; 7 Institute of Biology Valrose, University of Sophia-Antipolis, Nice, France.

**Keywords:** Inflammation, Pulmonology, Apoptosis, Extracellular matrix, Fibrosis

## Abstract

Idiopathic pulmonary fibrosis (IPF) is a fatal fibrotic lung disease characterized by impaired fibroblast clearance and excessive extracellular matrix (ECM) protein production. Wilms tumor 1 (WT1), a transcription factor, is selectively upregulated in IPF fibroblasts. However, the mechanisms by which WT1 contributes to fibroblast accumulation and ECM production remain unknown. Here, we investigated the heterogeneity of WT1-expressing mesenchymal cells using single-nucleus RNA-Seq of distal lung tissues from patients with IPF and control donors. WT1 was selectively upregulated in a subset of IPF fibroblasts that coexpressed several prosurvival and ECM genes. The results of both loss-of-function and gain-of-function studies were consistent with a role for WT1 as a positive regulator of prosurvival genes to impair apoptotic clearance and promote ECM production. Fibroblast-specific overexpression of WT1 augmented fibroproliferation, myofibroblast accumulation, and ECM production during bleomycin-induced pulmonary fibrosis in young and aged mice. Together, these findings suggest that targeting WT1 is a promising strategy for attenuating fibroblast expansion and ECM production during fibrogenesis.

## Abstract 2

Pulmonary fibrosis is a deadly lung disease marked by excess scar tissue buildup. We found that WT1 promotes myofibroblast accumulation involved in scar tissue formation.

## Graphical abstract

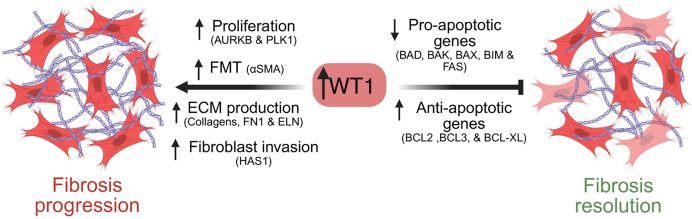

## Introduction

Pulmonary fibrosis is the final common pathologic pathway of a variety of acute and chronic lung injuries that are associated with dysregulated healing responses that include myofibroblast accumulation and aberrant lung regeneration (1). Despite its clinical and public health relevance, the pathophysiology of pulmonary fibrosis remains incompletely understood. However, it is often associated with fibroproliferation, fibroblast-to-myofibroblast transformation (FMT), and impaired apoptotic clearance of (myo)fibroblasts (2, 3). These processes collectively result in excessive extracellular matrix (ECM) production and scar tissue formation in the lung parenchyma. Idiopathic pulmonary fibrosis (IPF) is perhaps the most severe and enigmatic form of interstitial lung disease, and recent epidemiological studies suggest that the prevalence of the disease is increasing in the United States and globally (4, 5). The median survival after diagnosis is poor, typically in the range of 3–5 years (6). The mortality rate of IPF continues to rise each year, particularly among older adults with a history of environmental and occupational exposures (7). It remains one of the most rapidly progressive and fatal forms of interstitial lung disease in the aging population (8). The advent of 2 FDA-approved therapies for IPF has been a welcome advance for the field, but enthusiasm is tempered by their suppressive rather than remission-inducing effects and their formidable side effect profiles (3, 9, 10). While IPF pathogenesis is extensively studied, the molecular processes underlying impaired fibroblast clearance and ECM production remain largely unknown.

Wilms tumor 1 (WT1), a zinc-finger transcription factor, is a positive regulator of fibroproliferation that promotes excessive collagen and other ECM production in IPF fibroblasts. Several published studies have shown that WT1 is oncogenic in Wilms tumors and hematological malignancies (11, 12). WT1 is expressed at high levels in leukemic blast cells, increasing progenitor cell proliferation and survival (13, 14). WT1 is expressed during lung development in most mesothelial cells (and not in myofibroblasts) (3), where it is thought to mediate transformation into fibroblasts and smooth muscle cells in a process called mesothelial-mesenchymal transformation (MMT) (3, 15–17). During postnatal and adult stages of lung growth, there is little or no expression of WT1, and MMT does not occur (3, 15, 16, 18). Supporting the critical role for WT1 in lung development and other critical organs such as the heart, homozygous WT1-mutant mice die between E13.5 and E14.5; however, heterozygous WT1-mutant mice with reduced expression of WT1 are viable, fertile, and normal in size (15). WT1 is overexpressed in the distal lung fibroblasts and mesothelial cells in IPF (15, 16, 18). Similarly, in a mouse model of TGF-α–induced pulmonary fibrosis, WT1^+^ myofibroblasts have been shown to accumulate in thickened subpleural fibrotic lesions due to transformation of fibroblasts to myofibroblasts through MMT (3, 15, 18). WT1 expression is also elevated in fibroblasts from other organs undergoing fibrotic remodeling (19–21). Several recent studies have shown that WT1 induces proliferation, MMT, and ECM production in fibroblasts and that the loss of 1 allele is sufficient to attenuate both TGF-α– and bleomycin-induced pulmonary fibrosis (3). However, the mechanisms by which WT1 contributes to myofibroblast accumulation in pulmonary fibrosis have remained unclear.

In this study, we examined the role of WT1 in (myo)fibroblast survival and ECM production through the use of single-nucleus RNA-Seq (snRNA-Seq) and fibroblast-specific gain-of-function and loss-of-function mouse models. Our findings identified WT1 as a mediator of fibroblast dysfunction and a potential therapeutic target for fibrosing lung diseases.

## Results

### snRNA-Seq of the distal lung cells in normal and IPF lungs.

**Figure 1:**
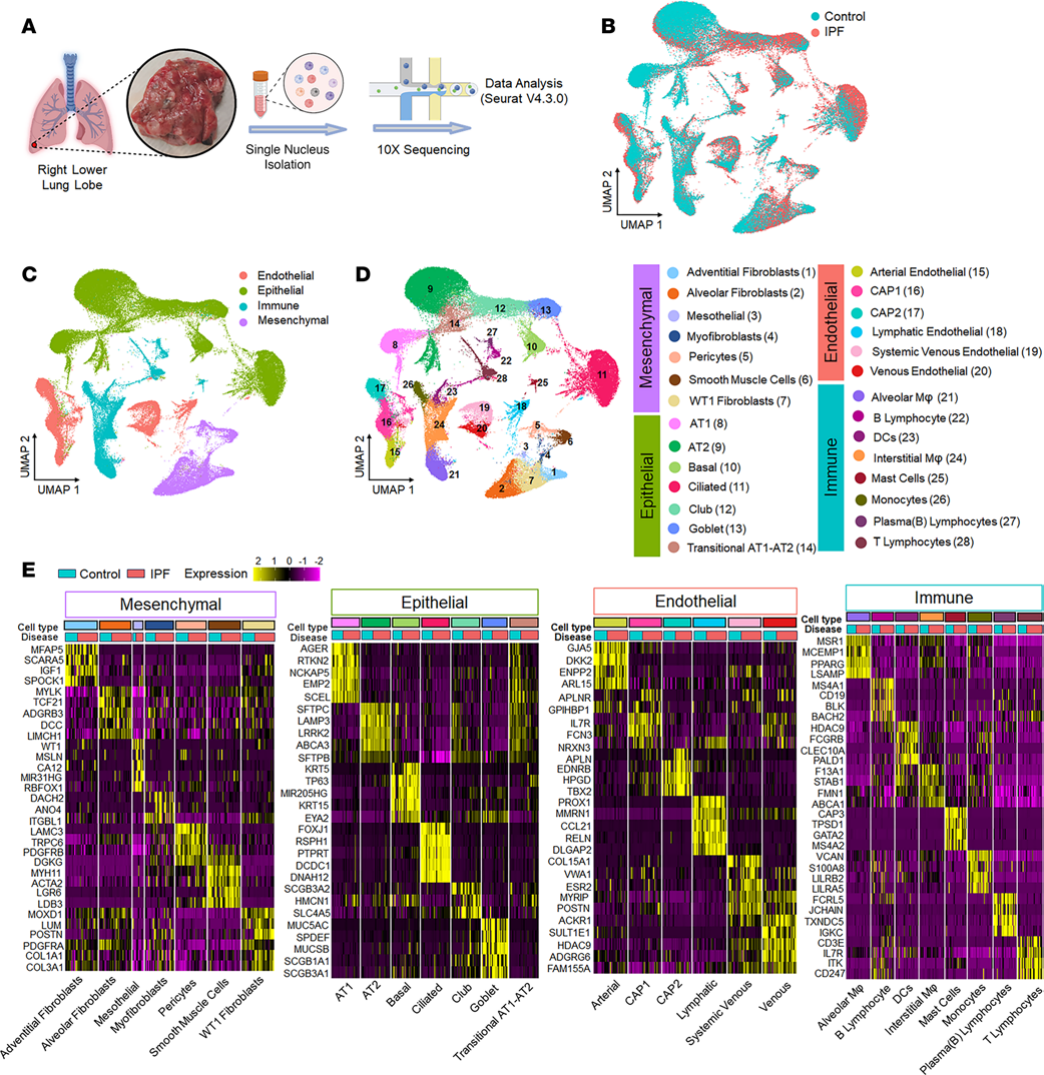
snRNA-Seq of lung cells in the distal regions of normal and IPF lungs. (**A**) Schematic of lung single-nucleus sample preparation and the workflow for snRNA sequencing and analysis. (**B**) UMAP plot of all cells colored according to IPF (*n* = 18) and control donor (*n* = 11) lung samples. Each dot on the UMAP represents a nucleus. (**C**) UMAP plot of 4 major cell lineages colored to denote epithelial, endothelial, mesenchymal, and immune cell types. (**D**) UMAP plot of all 28 unique cell subpopulations identified. (**E**) Heatmaps showing marker gene expression across 28 identified cell types, grouped into 4 major lineages. Each row represents gene expression across cell types from all samples, whereas each column shows the average gene expression per individual, grouped by disease condition. Only genes with an adjusted *P* value of less than 0.05 (Wilcoxon rank-sum test) are shown. Scaled gene expression values are used for visualization.

**Figure 2:**
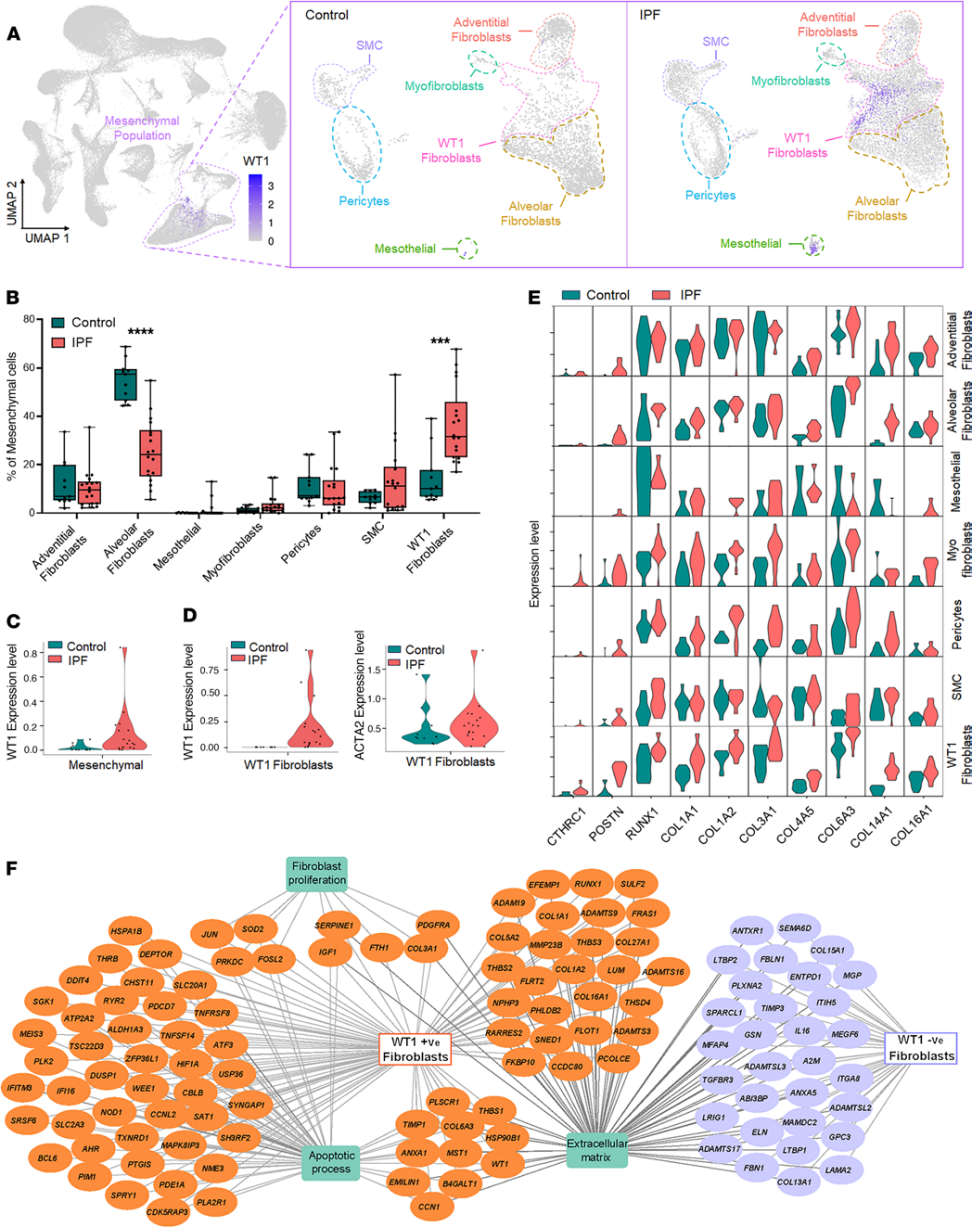
Fibroblast heterogeneity and identification of WT1 fibroblasts in the distal regions of IPF lungs. (**A**) UMAP plots showing normalized WT1 gene expression for all cells and further subdivided by IPF and control donor lung samples with 7 mesenchymal cell types. Color intensity reflects expression levels, with darker shades indicating higher expression. (**B**) Boxplots showing the proportion of each cell type within mesenchymal cell types across IPF and control donor lung samples. Each dot represents an individual sample, and the proportions sum to 100% per sample. ***P < 0.001 and ****P < 0.0001, by multiple 2-tailed Student’s *t* tests. (**C**) Violin plot showing the average WT1 expression in the mesenchymal cell population, compared between control and IPF samples. Each dot represents an individual sample. *P* < 0.05, by Wilcoxon rank-sum test. (**D**) Violin plot showing the average WT1 expression in the WT1 fibroblast population, compared between control and IPF samples. Each dot represents an individual sample. *P* < 0.05, by Wilcoxon rank-sum test. (**E**) Violin plots showing the average expression of fibrosis-associated genes (*CTHRC1*, *POSTN, RUNX1*, and collagen genes) across all mesenchymal cell subpopulations, comparing control and IPF samples. (**F**) DEGs in WT1-positive cells and WT1-negative cells of the WT1 fibroblast population were analyzed using ToppFun and visualized using CytoScape. Orange- and purple-colored circles represent genes that were upregulated in WT1-positive cells and WT1-negative cells, respectively. The green-colored boxes represent enriched biological processes for the DEGs.

To gain unbiased insight into WT1 expression dynamics and cell-type specificity in human lungs, we performed snRNA-Seq using the 10X Genomics platform to profile nuclei from 18 IPF and 11 normal donor lung samples, isolated from the distal regions of the right lower lobe (Figure 1A and Supplemental Table 1; supplemental material available online with this article; https://doi.org/10.1172/JCI188819DS1). After randomization of samples during library preparation and sequencing to minimize batch effects, followed by doublet removal and cell and sample quality control (QC), we obtained a total of 100,058 single-nucleus transcriptomes from 29 QC-passed samples, including 33,057 lung nuclei from control subjects and 67001 lung nuclei from IPF patients using Seurat (version 4.3.0) in R studio (Supplemental Table 1). We performed preprocessing, integration, and clustering using reciprocal principal component analysis (rPCA) integrative analysis, a more conservative approach that preserves distinctions between cells in different biological states. All clusters had acceptable QC metrics, and no cluster was composed of nuclei captured only from individual patients, samples, or technical covariates, indicating that the data integration was successful and showed disease-related heterogeneity (Figure 1B and Supplemental Figure 1). This captured all major cell types of the lung, which were identified using the canonical markers *COL1A2* for mesenchymal cells, *PECAM1* for endothelial cells, *EPCAM* for epithelial cells, and *PTPRC* for immune cells (Figure 1C and Supplemental Figure 2). Using unsupervised clustering and canonical markers, we identified 28 unique cell subtype populations, including mesenchymal cells (7), epithelial cells (7), endothelial cells (6), and immune cells (8) (Supplemental Table 2 and Figure 1D). The heatmaps depict the marker gene expression pattern for each major cell type and individual, supporting the validity of our clustering and annotations (Figure 1E). Among the 4 major cell types, we observed an overall increase in the proportion of mesenchymal, epithelial, and endothelial cells compared with immune cells (22, 23), highlighting the effectiveness of single-nucleus isolation in capturing parenchymal cell types that are traditionally more difficult than immune cells to extract as single cells from tissues (Supplemental Figure 3). Within the mesenchymal cells, we identified 7 subtypes, including alveolar fibroblasts, adventitial fibroblasts, myofibroblasts, pericytes, smooth muscle cells (SMCs), mesothelial cells, and WT1 fibroblasts (Figure 2, A and B, and Supplemental Figures 4 and 5). Among the major mesenchymal cell populations, we observed an increased proportion of WT1 fibroblasts, accompanied by a decrease in the proportion of alveolar fibroblasts, in the distal regions of IPF lungs compared with control samples (Figure 2, A and B). WT1 expression was elevated in mesenchymal samples of IPF, particularly in the WT1 fibroblast subpopulation, a trend also observed in other scRNA-Seq datasets (Figure 2, C and D, and Supplemental Figure 6). WT1 fibroblasts expressed high levels of *CTHRC1*, *POSTN*, *RUNX1*, and several collagen genes, including *COL1A1*, *COL1A2*, *COL3A1*, *COL4A5*, *COL6A3*, *COL14A1*, and *COL16A1* in patients with IPF compared with control individuals (Figure 2E). While WT1 fibroblasts were found only in samples from patients with IPF, they were composed of both WT1^+^ and WT^–^ fibroblasts. To further investigate the effects of WT1 expression on fibrosis-associated gene expression, we assessed differentially expressed genes in WT1^+^ and WT1^–^ fibroblasts compared with other mesenchymal cells in samples from patients with IPF and visualized the major gene networks using gene function enrichment analysis. Notably, we observed that genes were associated with fibroblast proliferation and apoptosis as the key biological processes enriched in WT1^+^ fibroblasts compared with WT1^–^ fibroblasts (Figure 2F). Both WT1^+^ and WT1^–^ fibroblasts were enriched for ECM genes compared with other mesenchymal cells in individuals with IPF. Collectively, these observations indicate that WT1^+^ fibroblasts represent a pathogenic mesenchymal cell population in the distal lung in IPF.

### WT1 is upregulated in distal lung mesenchymal cells in IPF.

**Figure 3:**
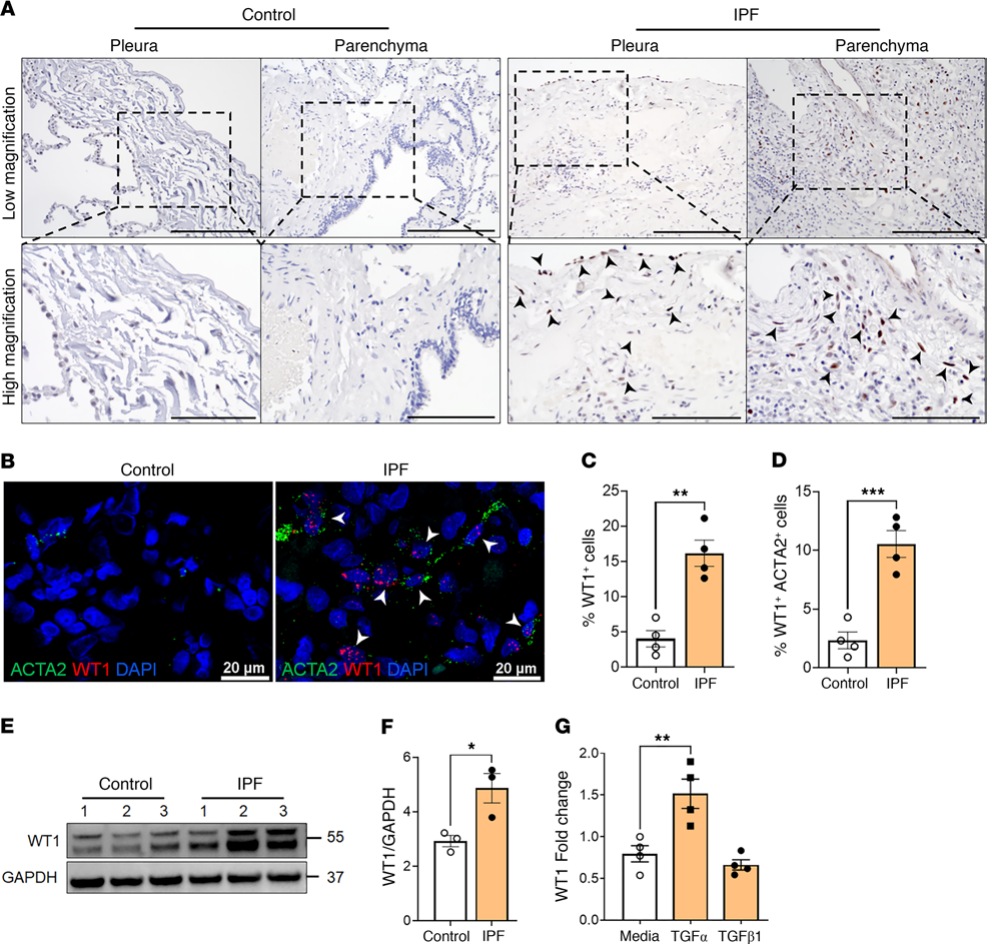
WT1 is upregulated in IPF distal lung mesenchymal cells. (**A**) Immunostaining was performed with the anti-WT1 antibody on lung sections from control and individuals with IPF. Representative images of pleural and parenchymal regions were obtained at low (original magnification, ×20; scale bar: 200 μm) and high (original magnification, ×40; scale bar: 100 μm) magnification. Arrowheads highlight WT1 staining on pleural mesothelial cells and spindle-shaped fibroblasts in the distal fibrotic lesions of lung parenchyma. (**B**) Representative fluorescence confocal RNA-ISH images showing *WT1* (red) and *ACTA2* (green) expression in control and IPF lung tissues. White arrowheads illustrate the double-positive cells. Scale bars: 20 μm. (**C**) Quantification of WT1^+^ cells normalized to total lung cells in lung section images from IPF and control lungs (*n* = 4/group). **P < 0.01, by 2-tailed Student’s *t* test. (**D**) Quantification of mesenchymal cells double-positive for WT1 and ACTA2 among total lung cells in lung section images from IPF and control lungs (*n* = 4/group). ***P < 0.001, by 2-tailed Student’s *t* test. (**E**) Primary lung-resident fibroblasts isolated from lung cultures of control and IPF lungs were immunoblotted with antibodies against WT1 and GAPDH. (**F**) WT1 protein levels were normalized to GAPDH and are shown as fold change (*n* = 3/group). *P < 0.05, by 2-tailed Student’s *t* test. (**G**) *WT1* transcripts were measured by RT-PCR in normal lung fibroblasts treated with media, TGF-α (50 ng/mL), or TGF-β1 (20 ng/mL) for 16 hours (*n* = 4/group). **P < 0.01, by 1-way ANOVA.

To validate the accumulation of WT1-expressing mesenchymal cells in the distal regions of IPF lungs, we performed immunostaining for WT1 in the distal lung sections of control donors and patients with IPF. Immunostaining for WT1 supported earlier findings that WT1 is selectively upregulated in mesothelial cells and spindle-shaped mesenchymal cells in fibrotic lesions of pleura and distal lung parenchyma of IPF lungs; however, there was limited or no staining observed in either mesothelial or other lung cells in control individuals (Figure 3A and Supplemental Figure 7).

To further validate WT1 upregulation in fibroblasts of IPF lungs, we conducted RNA ISH for WT1 and ACTA2, a well-established myofibroblast marker, in control and IPF lung tissues. Consistent with our snRNA-Seq findings, we observed a significant increase in mesenchymal cells costaining for both WT1 and ACTA2 in distal lung regions of IPF, but not control, donor lung samples (Figure 3B and Supplemental Videos 1–3). Quantitative analysis confirmed a marked increase in WT1^+^ cells and in cells positive for both WT1 and ACTA2 in IPF samples, supporting their selective accumulation in distal fibrotic lung lesions from individuals with IPF (Figure 3, C and D).

To show that WT1 was upregulated in IPF mesenchymal cells, we measured WT1 protein expression in fibroblasts isolated from IPF and control lungs (Figure 3, E and F). Indeed, we observed a marked increase in WT1 protein levels in IPF fibroblasts compared with fibroblasts isolated from control lungs (Figure 3, E and F). Given that TGF-β and TGF-α are known positive regulators of fibroblast activation in the pathogenesis of pulmonary fibrosis (24, 25), we sought to identify the potential role of these growth factors in the upregulation of WT1 in fibroblasts. We treated normal human fibroblasts with media, TGF-β1, or TGF-α for 16 hours in low-serum conditions and observed elevated *WT1* transcript levels in fibroblasts treated with TGF-α but not TGF-β1 compared with media-treated control fibroblasts (Figure 3G). Taken together, these findings suggest that WT1 was upregulated in IPF mesenchymal cells and that TGF-α functioned as a positive regulator of WT1 expression in lung mesenchymal cells.

### WT1 is a positive regulator of cell survival and ECM gene expression in IPF fibroblasts.

**Figure 4:**
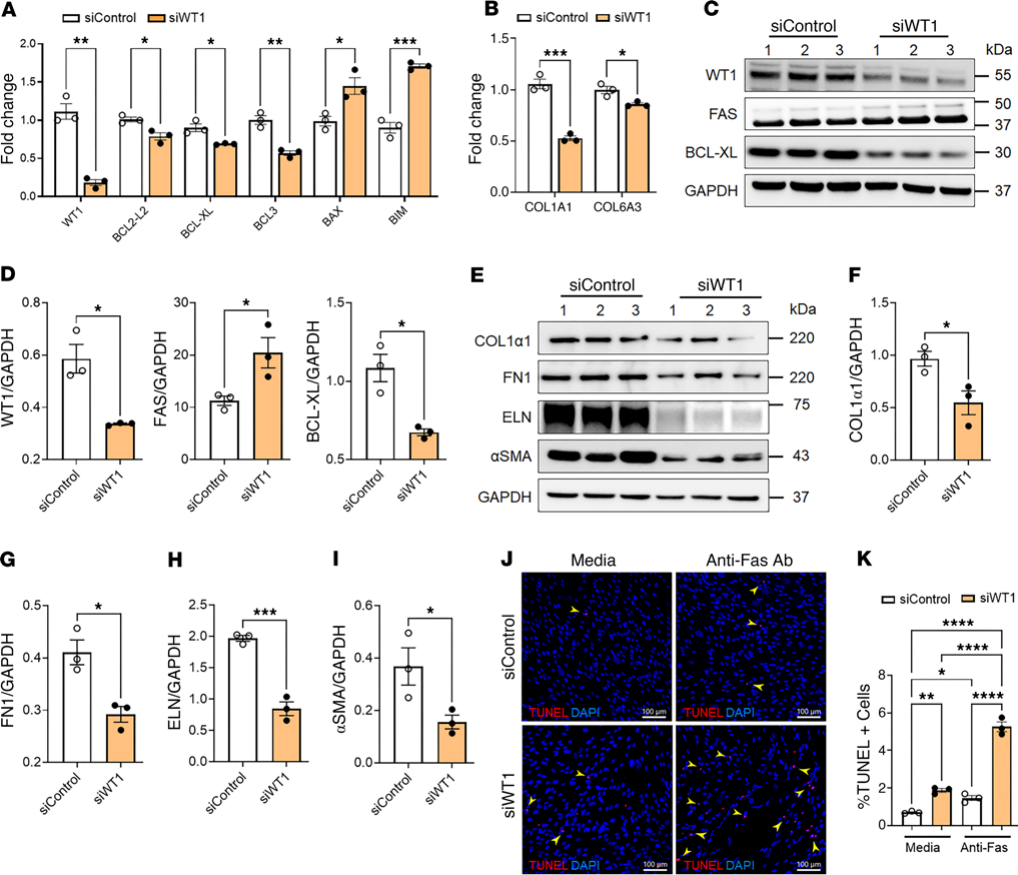
Loss of WT1 attenuates the expression of genes involved in fibroblast survival and ECM production. (**A**) Quantification of both proapoptotic (*BAX* and *BIM*) and antiapoptotic (*BCL2-L2*, *BCL-XL*, and *BCL3*) gene transcripts using RT-PCR in IPF fibroblasts treated with either control or WT1 specific siRNA for 72 hours. Multiple unpaired, 2-tailed Student’s *t* tests were used for comparisons (*n* = 3/group). *P < 0.05, **P < 0.01, and ***P < 0.001, by multiple unpaired, 2-tailed Student’s *t* tests. (**B**) Quantification of collagen gene transcripts (*COL1A1* and *COL6A3*) using RT-PCR in IPF fibroblasts treated with either control or WT1-specific siRNA for 72 hours (*n* = 3/group). *P < 0.05, and ***P < 0.001, by multiple unpaired, 2-tailed Student’s *t* tests. (**C** and **D**) IPF fibroblasts were treated with either control or WT1-specific siRNA for 72 hours, and cell lysates were immunoblotted with antibodies against WT1, FAS, BCL-XL, or GAPDH. Quantification of WT1, FAS, and BCL-XL protein levels normalized to GAPDH (*n* = 3/group). *P < 0.05, by 2-tailed Student’s *t* test. (**E**–**I**) IPF fibroblasts were treated with either control or WT1-specific siRNA for 72 hours, and cell lysates were immunoblotted with antibodies against COL1α1, FN1, ELN, αSMA, or GAPDH. Quantification of COL1α1, FN1, ELN, and αSMA protein levels normalized to GAPDH (*n* = 3/group). *P < 0.05 and ***P < 0.001, by 2-tailed Student’s *t* test. (**J**) IPF fibroblasts were treated with either control or WT1-specific siRNA for 48 hours, followed by anti-Fas antibody treatment for another 24 hours, and cells were stained to quantify total TUNEL^+^ cells. Representative confocal images were obtained at ×20 original magnification. Scale bars: 100 μm. (**K**) The percentage of TUNEL^+^ (red color) cells in the total DAPI-stained (blue color) cells was quantified using MetaMorph image analysis. One-way ANOVA was used for comparisons (*n* = 3/group). *P < 0.05, **P < 0.01, and ****P < 0.0001, by 1-way ANOVA for comparisons.

To investigate the effects of WT1 on fibroblast survival, we knocked down WT1 in IPF fibroblasts isolated from the distal fibrotic lung lesions. As shown in Figure 4A, WT1 knockdown led to a significant decrease in the expression of antiapoptotic genes, including *BCL2-L2*, *BCL-XL*, and *BCL3*, while concurrently increasing the expression of key proapoptotic genes such as *BAX* and *BIM*, consistent with a role for WT1 in promoting fibroblast survival by modulating apoptotic pathways. Knockdown of WT1 also resulted in the downregulation of ECM genes, specifically *COL1A1* and *COL6A3*, in fibroblasts treated with WT1-specific siRNA compared with controls treated with nontargeting siRNA (Figure 4B). To validate the effect of WT1 deficiency on proapoptotic gene expression, we conducted Western blot analysis, which revealed a significant upregulation of the apoptosis-inducing protein FAS in IPF fibroblasts following WT1 knockdown (Figure 4, C and D). Conversely, expression of the antiapoptotic protein BCL-XL was markedly reduced in WT1-deficient IPF fibroblasts compared with those treated with control siRNA, further confirming the shift toward a proapoptotic state in response to WT1 knockdown (Figure 4, C and D). Furthermore, Western blot analysis demonstrated a significant reduction in the levels of ECM proteins, including COL1α1, FN1, ELN, and α smooth muscle actin (αSMA), in IPF fibroblasts treated with WT1-specific siRNA compared with control siRNA (Figure 4, E–I). Also, we assessed the effects of WT1 knockdown on collagen secretion and found a significant reduction in secreted collagen proteins in conditioned media from IPF fibroblasts treated with WT1-specific siRNA compared with IPF fibroblasts treated with control siRNA (Supplemental Figure 8). These findings highlight the pivotal role of WT1 in supporting fibroblast survival and ECM production, key processes in the pathogenesis of lung fibrosis. To further elucidate the role of WT1 in the impaired clearance of IPF fibroblasts, we conducted TUNEL assays on IPF fibroblasts treated with either control siRNA or WT1-specific siRNA for 72 hours. WT1 knockdown significantly increased the number of TUNEL^+^ fibroblasts under basal conditions and following stimulation with an anti-Fas antibody compared with cells treated with control siRNA (Figure 4, J and K). These results suggest that WT1 plays a pivotal role in the persistence of IPF fibroblasts by protecting them against apoptotic signals.

### WT1 overexpression promotes fibroblast survival and ECM production in normal fibroblasts.

**Figure 5:**
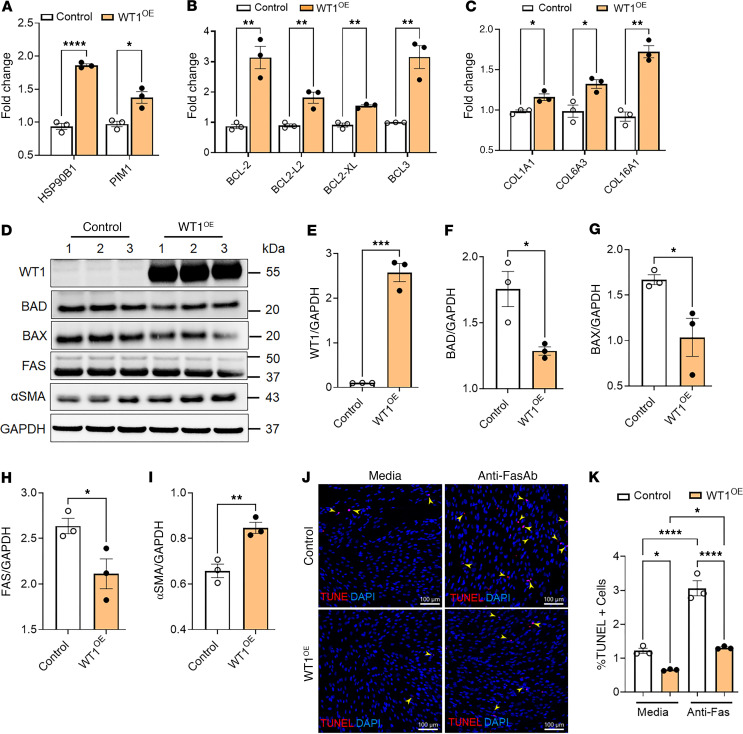
WT1 functions as a positive regulator of lung-resident fibroblast survival. (**A**) Quantification of prosurvival gene (*HSP90B1*, and *PIM1*) transcripts using RT-PCR in normal lung fibroblasts transduced with either control or WT1-overexpressing adenoviral particles for 72 hours (*n* = 3/group). *P < 0.05 and ****P < 0.0001, by multiple unpaired, 2-tailed Student’s *t* tests for comparisons. (**B**) Normal lung fibroblasts were transduced with either control or WT1-overexpressing adenoviral particles for 72 hours, and prosurvival gene (*BCL2*, *BCL2-L2*, *BCL-XL*, and *BCL3*) transcript levels were quantified by RT-PCR (*n* = 3/group). **P < 0.01, by multiple unpaired, 2-tailed Student’s *t* tests. (**C**) Normal lung fibroblasts were transduced with either control or WT1-overexpressing adenoviral particles for 72 hours, and ECM-associated gene (*COL1A1*, *COL6A3*, and *COL16A1*) transcript levels were quantified by RT-PCR (*n* = 3/group). *P < 0.05 and **P < 0.01, by multiple unpaired, 2-tailed Student’s *t* tests. (**D**–**I**) Normal lung fibroblasts were transduced with either control or WT1-overexpressing adenoviral particles for 72 hours, and cell lysates were immunoblotted with antibodies against WT1, BAD, BAX, FAS, αSMA, or GAPDH. Quantification of WT1, BAD, BAX, FAS, and αSMA protein levels normalized to GAPDH (*n* = 3/group). *P < 0.05, **P < 0.01, and ***P < 0.001, by 2-tailed Student’s *t* test. (**J**) Normal lung fibroblasts were treated with either control or WT1-overexpressing adenoviral particles for 48 hours, followed by anti-Fas antibody treatment for another 24 hours, and fibroblasts were stained for TUNEL (red). Representative confocal images are shown; nuclei were stained with DAPI (blue). Original magnification, ×20. Scale bars: 100 μm. Yellow arrowheads highlight TUNEL^+^ apoptotic cells. (**K**) Quantification of TUNEL^+^ fibroblasts using MetaMorph image analysis (*n* = 3/group). *P < 0.05 and ****P < 0.0001, by 1-way ANOVA.

To determine whether WT1 overexpression alone can promote survival and ECM gene expression, we treated normal lung fibroblasts with either a control adenovirus or a WT1-overexpressing adenovirus for 72 hours. As expected, we observed a significant increase in WT1 transcript levels in fibroblasts infected with the WT1-expressing adenovirus compared with the control adenovirus (Supplemental Figure 9). To examine the effects of WT1 overexpression, we selected a panel of prosurvival genes that are differentially expressed in WT1^+^ myofibroblasts in our snRNA-Seq datasets. We confirmed that overexpression of WT1 induced the expression of prosurvival genes, including *HSP90B1* and *PIM1* (Figure 5A). Similarly, WT1 overexpression in normal lung fibroblasts resulted in a marked upregulation of antiapoptotic genes, including *BCL2*, *BCL2-L2*, *BCL-XL*, and *BCL3,* mirroring the inverse effects observed in WT1 loss-of-function studies in IPF fibroblasts (Figure 5B and Figure 4A). Also, WT1 overexpression resulted in a significant increase in the expression of several ECM-associated gene transcripts, including *COL1A1*, *COL6A3*, and *COL16A1* (Figure 5C). We next confirmed the effects of WT1 overexpression on BAD, BAX, FAS, and αSMA protein levels by Western blot analysis of normal fibroblasts infected with WT1-overexpressing adenovirus compared with control adenovirus for 72 hours. We observed a significant decrease in the expression of the proapoptotic proteins BAD, BAX, and FAS and a significant increase in αSMA levels in WT1-overexpressing fibroblasts (Figure 5, D–I). To determine whether WT1 induces fibroblast survival, we performed TUNEL assays in normal fibroblasts treated with either control adenovirus or WT1-overexpressing adenovirus for 72 hours. WT1 overexpression in normal fibroblasts resulted in a significant decrease in TUNEL^+^ cells upon treatment with anti-Fas antibody compared with control (Figure 5, J and K). Collectively, these findings suggest that WT1 functioned as a negative regulator of fibroblast apoptosis, consistent with a role for WT1 in fibroblast survival, accumulation, and excess ECM production in the pathogenesis of pulmonary fibrosis.

### WT1 induces survival gene expression in mouse lung fibroblasts.

**Figure 6:**
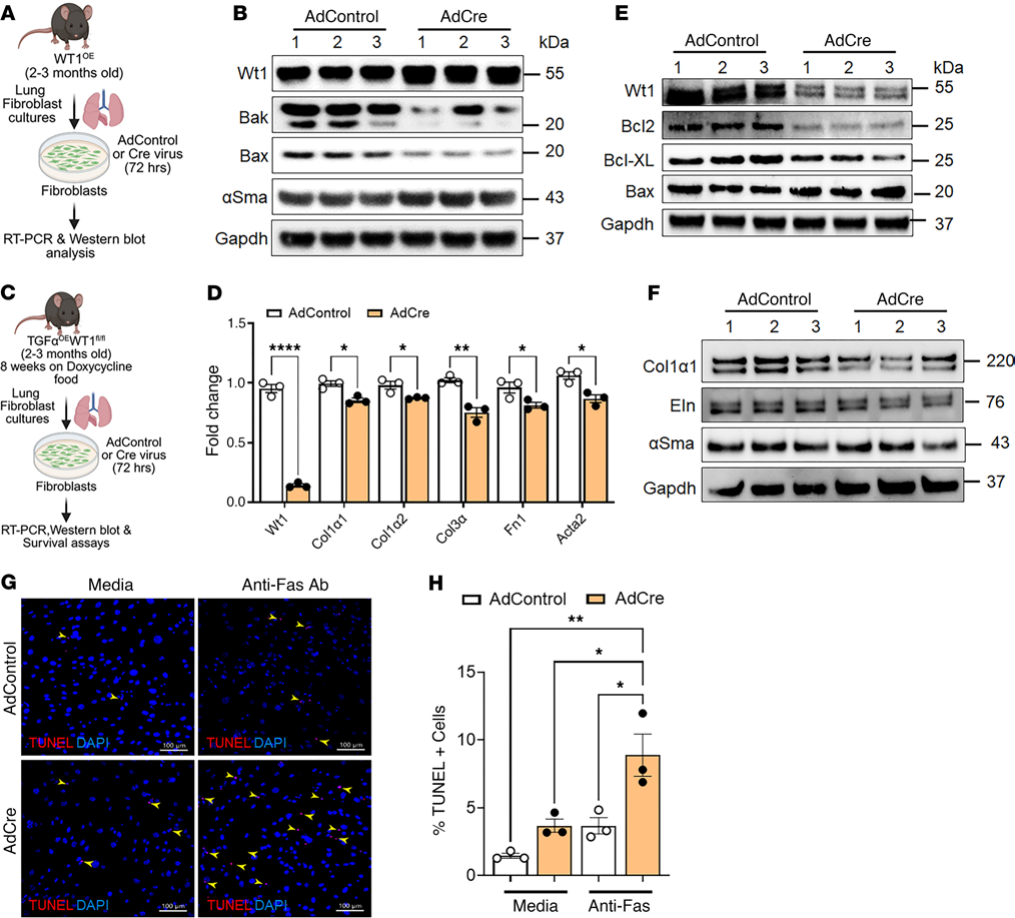
Overexpression of WT1 augments prosurvival gene expression in mouse lung resident fibroblasts. (**A**) Schematic workflow for **B** to **C**, illustrating the isolation of fibroblasts from WT1^OE^ mouse lung cultures. Fibroblasts were infected with either control or Cre-expressing adenovirus for 72 hours to induce WT1 overexpression. (**B**) Fibroblasts were infected with either control adenovirus (AdControl) or Cre-expressing adenovirus (AdCre) for 72 hours, and cell lysates were immunoblotted with antibodies against Wt1, Bak, Bax, αSma, or Gapdh. (**C**) Schematic workflow for **E** to **I**, illustrating the isolation of fibroblasts from lung cultures derived from *TGF-α^OE^WT1^fl/fl^* mice on doxycycline-treated food for 8 weeks. Lung-resident fibroblasts were infected with either control or Cre-expressing adenovirus for 72 hours to delete WT1. (**D**) RT-PCR quantification of WT1 and ECM gene transcript expression, including *Col1α1*, *Col1α2*, *Col3α*, *Fn1*, and *Acta2*, in WT1-deficient fibroblasts compared with control fibroblasts (*n* = 3/group). *P < 0.05, **P < 0.01, and ****P < 0.0001, by multiple unpaired, 2-tailed Student’s *t* tests. (**E** and **F**) Lung-resident fibroblasts isolated from lung cultures of *TGF-α^OE^ WT1^fl/fl^* mice on doxycycline-treated food for 8 weeks were infected with either control or Cre-expressing adenovirus for 72 hours, and cell lysates were immunoblotted with antibodies against Wt1, Bcl2, Bcl-XL, Bax, Col1α, Eln, Fn1, αSma, or Gapdh. (**G**) Fibroblasts were infected with either control or Cre-expressing adenovirus for 48 hours, followed by treatment with anti-Fas for an additional 24 hours. Cells were then stained to quantify TUNEL^+^ apoptotic cells (yellow arrowheads). Representative confocal images are shown. Original magnification, ×20. Scale bars: 100 μm. (**H**) Quantification of the percentage of TUNEL^+^ cells (red) in total cells stained with DAPI (blue) was performed using MetaMorph image analysis (*n* = 3/group). *P < 0.05 and **P < 0.01, by 1-way ANOVA.

To elucidate the role of WT1 in the survival of lung-resident fibroblasts, we generated fibroblasts from lung cultures of Cre-inducible WT1-overexpressing (WT1^OE^) transgenic mice and infected them with control adenovirus or Cre-expressing adenovirus for 72 hours (Figure 6A). As shown in Figure 6B, overexpression of WT1 led to a reduction in protein levels of the proapoptotic markers Bak and Bax, while notably increasing the expression of αSMA compared with control fibroblasts (Figure 6B and Supplemental Figure 10A), suggesting a role for WT1 in promoting fibroblast survival and myofibroblast differentiation.

To examine whether WT1 is required for the maintenance of fibroblast activation, we isolated activated fibroblasts from lung cultures of fibrotic lung lesions from *TGF-a^OE^* *WT1^fl/fl^* mice on doxycycline for 8 weeks (Figure 6C). We selectively deleted WT1 in activated fibroblasts by treating them with Cre-expressing adenovirus and assessed the changes in survival, ECM gene expression, and apoptosis. As expected, we observed a loss of Wt1 expression in activated fibroblasts by treating them with Cre-expressing adenovirus compared with fibroblasts treated with control adenovirus for 72 hours (Figure 6D). Knockdown of WT1 resulted in reduced expression of ECM genes, including *Col1a1*, *Col1a2*, *Col3a*, *Fn1*, and *Acta2* in activated fibroblasts infected with Cre-expressing adenovirus compared with those infected with control adenovirus (Figure 6D). Western blot analysis showed that knockdown of WT1 resulted in a significant decrease in survival-associated proteins, including Bcl2 and Bcl-XL, while it upregulated the expression of the proapoptotic Bax protein (Figure 6E and Supplemental Figure 10B). Similarly, Wt1 knockdown led to a significant reduction in Col1α1, elastin, and αSma protein expression levels compared with control cells (Figure 6F and Supplemental Figure 10C). Additionally, the TUNEL assays demonstrated that WT1 knockdown increased the number of TUNEL^+^ fibroblasts in the presence of anti-Fas antibodies, further supporting this finding (Figure 6, G and H). Thus, our findings provide complementary evidence that WT1 is required for maintenance of the profibrotic functions of activated fibroblasts in fibrotic lung lesions.

### Overexpression of WT1 in fibroblasts augments bleomycin-induced pulmonary fibrosis.

To assess the direct effects of WT1 overexpression on fibroblasts, we bred *PDGFRα^CreERT^* mice with *WT1^OE^* mice to generate fibroblast-specific conditional WT1-overexpressing (cWT1^OE^) mice and control (PDGFRα^CreERT^) mice. Fourteen-week-old cWT1^OE^ and control mice were treated with 2 tamoxifen injections per week for 2 weeks. We observed no significant changes in lung architecture or collagen abundance in Masson’s trichrome–stained lung sections from control or cWT1^OE^ mice treated with tamoxifen (Supplemental Figure 11A), and there were no differences in total lung hydroxyproline levels (Supplemental Figure 11B). To assess the upregulation of WT1 and fibrosis-associated genes, we quantified transcript levels of WT1, ECM, and apoptosis-associated genes in total lung RNA from tamoxifen-treated control and cWT1^OE^ mice. As expected, we found that WT1 expression was markedly increased in cWT1^OE^ mice compared with expression in controls. However, no significant differences were observed in the expression of ECM-related genes (*Col1a1*, *Col3a*, *Col5a*, and *Acta2*) or apoptosis-associated genes (*Bak1*, *Bax*, *Bcl2*, and *Bcl-XL*) between the 2 groups (Supplemental Figure 11, C–E).

**Figure 7:**
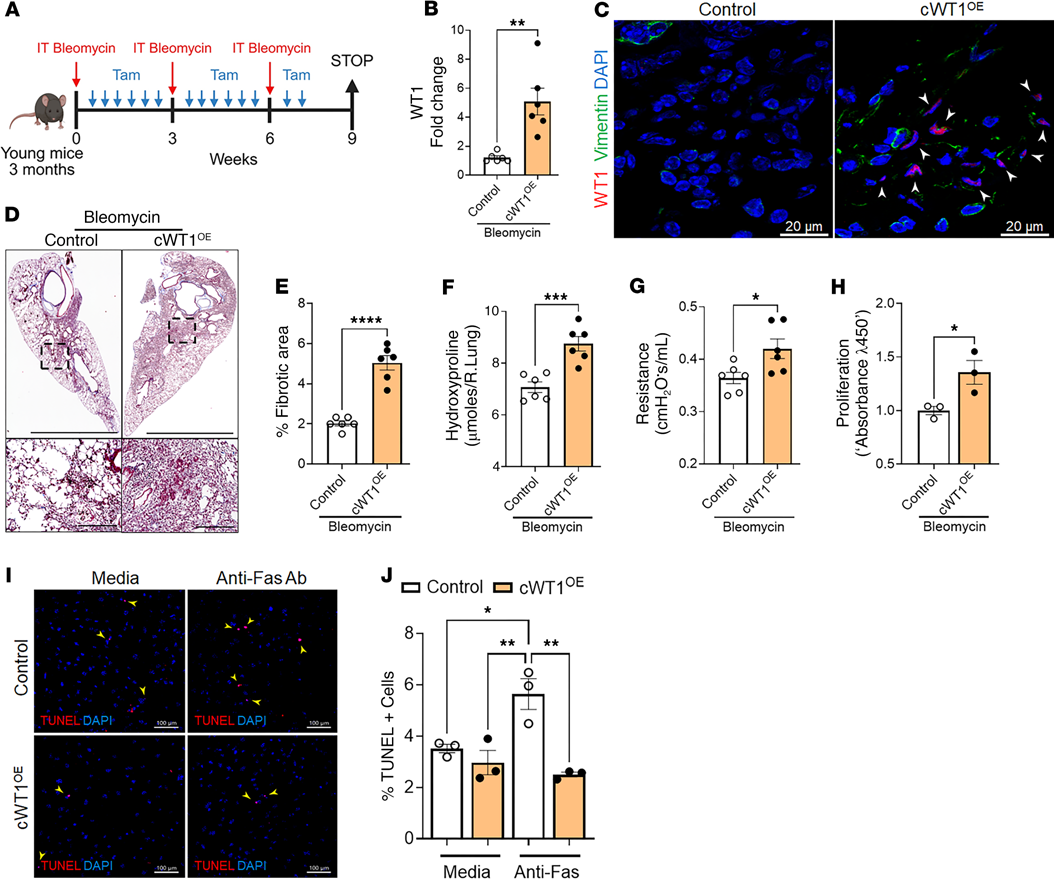
Fibroblast-specific WT1 overexpression augments bleomycin-induced pulmonary fibrosis in mice. (**A**) Schematic representation of the animal experiment involving *PDGFRα^CreERT^* (control) and *PDGFRα^CreERT^* *WT1^OE^* (cWT1^OE^) mice. Mice were treated repeatedly with bleomycin via intratracheal administration and with tamoxifen via intraperitoneal injection. (**B**) Quantification of *Wt1* gene transcripts by RT-PCR in total lung RNA isolated from control and cWT1^OE^ mice (*n* = 6/group). **P < 0.01, by 2-tailed Student’s *t* test. (**C**) Representative confocal images of lung sections from control and cWT1^OE^ mice co-immunostained for WT1 (red), vimentin (green), and DAPI (blue). Scale bars: 20 μm. (**D**) Representative images of Masson’s trichrome–stained lung sections from control and cWT1^OE^ mice. Original magnification, ×4 and ×20, respectively. Scale bars: 1,500 μm and 200 μm, respectively. (**E**) The percentage of fibrotic area was quantified in control and cWT1^OE^ mice using BZ-X image analysis (*n* = 6/group). ****P < 0.0001, by 2-tailed Student’s *t* test. (**F**) Hydroxyproline levels were measured in the right lungs of control and cWT1^OE^ mice (*n* = 6/group). ***P < 0.001, by 2-tailed Student’s *t* test. (**G**) Lung resistance was assessed using FlexiVent in control and cWT1^OE^ mice treated with bleomycin (*n* = 6/group). *P < 0.01, by 2-tailed Student’s *t* test. (**H**) Proliferation of fibroblasts was quantified using a BrdU incorporation assay in lung cultures from control and cWT1^OE^ mice treated with bleomycin (*n* = 3/group). *P < 0.05, by 2-tailed Student’s *t* test. (**I**) Fibroblasts from the lung cultures of control and cWT1^OE^ mice treated with bleomycin were treated with anti-Fas antibody for 24 hours, followed by TUNEL staining (red). Representative confocal images are shown; nuclei are stained with DAPI (blue). Original magnification, ×20. Scale bars: 100 μm. (**J**) Percentage of TUNEL^+^ fibroblasts in total DAPI^+^ fibroblasts (*n* = 3/group). *P < 0.05 and **P < 0.01, by 1-way ANOVA. Data are representative of 2 independent experiments with similar findings.

To investigate whether WT1 overexpression augments bleomycin-induced pulmonary fibrosis, control and cWT1^OE^ mice were treated with 2 tamoxifen injections per week for 7 weeks, and 3 intratracheal instillations of bleomycin at 3-week intervals for a total of 9 weeks (Figure 7A). We observed a significant increase in *WT1* transcript levels in total lung RNA of cWT1^OE^ mice compared with control mice treated with bleomycin (Figure 7B). To evaluate WT1 upregulation in fibroblasts, we coimmunostained lung sections with antibodies against WT1 and vimentin and observed WT1 upregulation in the fibroblasts of cWT1^OE^ mice compared with control mice treated with bleomycin (Figure 7C). Masson’s trichrome staining revealed a substantial increase in collagen staining in lung sections from the bleomycin-treated cWT1^OE^ group compared with sections from the bleomycin-treated control mice (Figure 7D). We observed a significant increase in the ratio of the fibrotic area to the total scanned area in the lung sections from cWT1^OE^ mice compared with those from control mice treated with bleomycin (Figure 7E). Importantly, total hydroxyproline levels were elevated in cWT1^OE^ mice compared with control mice treated with bleomycin (Figure 7F). Consistent with the increase in lung collagen levels, we observed a significant increase in lung resistance in cWT1^OE^ mice compared with control mice treated with bleomycin (Figure 7G). Next, we quantified the changes in ECM-associated gene expression in total lung transcripts of cWT1^OE^ mice compared with control mice treated with bleomycin. Consistent with the observed changes in collagen levels, we observed a significant increase in transcript levels of ECM genes, including *Col1a1*, *Col3a*, *Col5a*, *Eln*, and *Fn1*, in cWT1^OE^ mice compared with control mice treated with bleomycin (Supplemental Figure 12A). Consistent with the in vitro findings, we observed a significant decrease in the expression of proapoptotic genes, including *Bad* and *Bak1*, in cWT1^OE^ mice compared with control mice treated with bleomycin (Supplemental Figure 12B). Also, we observed elevated expression of the fibroproliferative gene *Plk1* in cWT1^OE^ mice compared with the control mice treated with bleomycin (Supplemental Figure 12C). To assess the effect of WT1 overexpression on fibroblast activation, we quantified the proliferative changes and apoptosis in lung fibroblasts isolated from lung cultures of cWT1^OE^ and control group mice treated with bleomycin. As assessed by BrdU incorporation, we observed a significant increase in the proliferation of fibroblasts from cWT1^OE^ mice compared with control mice treated with bleomycin (Figure 7H). Similarly, we observed reduced TUNEL^+^ cells with anti-Fas treatment of fibroblasts from cWT1^OE^ mice compared with cells from control mice treated with bleomycin (Figure 7, I and J). Taken together, our findings demonstrate the profibrotic effects of WT1 upregulation in promoting fibroblast proliferation, survival, and ECM production during bleomycin-induced pulmonary fibrosis.

### Overexpression of WT1 in fibroblasts augments pulmonary fibrosis in aged mice.

**Figure 8:**
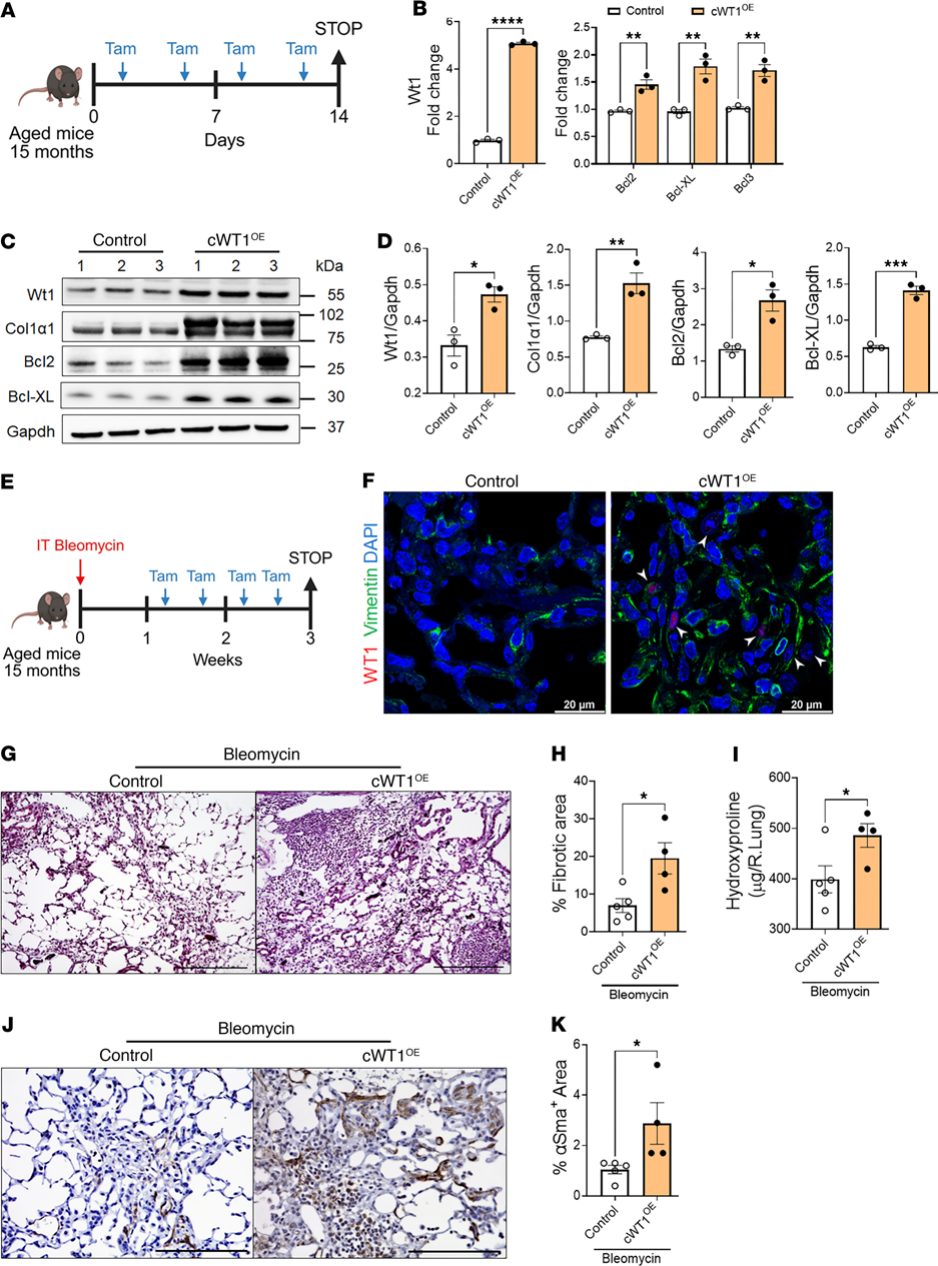
WT1 augments fibroblast survival and bleomycin-induced pulmonary fibrosis in aged mice. (**A**) Schematic presentation of the study using 15-month-old *PDGFRα^CreERT^* (control) and *PDGFRα^CreERT^* *WT1^OE^* (cWT1^OE^) mice. (**B**) Quantification of cell-survival gene transcripts (*Wt1*, *Bcl2*, *Bcl-XL*, and *Bcl3*) in total lung transcripts for control and cWT1^OE^ mice (*n* = 3/group). **P < 0.01 and ****P < 0.0001, by multiple unpaired, 2-tailed Student’s *t* test. (**C**) The total lung lysates were immunoblotted with antibodies against Wt1, Col1α1, Bax, Bcl2, Bcl-XL, and Gapdh for control and cWT1^OE^ mice. (**D**) Wt1, Col1α1, Bcl2, and Bcl-XL protein levels were normalized to Gapdh (*n* = 3/group). *P < 0.05, **P < 0.01, and ***P < 0.001, by 2-tailed Student’s *t* test. (**E**) Schematic representation of the animal experiment involving 15-month-old *PDGFRα^CreERT^* (control) and *PDGFRα^CreERT^* *WT1^OE^* (cWT1^OE^) mice. Mice were treated with bleomycin and tamoxifen as shown in the schema. (**F**) Representative confocal images of lung sections from 15-month-old control and cWT1^OE^ mice. Arrowheads highlight lung fibroblasts that costained for WT1 (red), vimentin (green), and DAPI (blue) in lung sections from control and cWT1^OE^ mice treated with bleomycin. Scale bars: 20 μm. (**G**) Representative images of Masson’s trichrome-stained lung sections from control and cWT1^OE^ mice treated with bleomycin. Scale bar: 200 μm. (**H**) The percentage of fibrotic area was quantified in control and cWT1^OE^ mice using BZ-X image analysis (*n* = 4–5/group). *P < 0.05, by 2-tailed Student’s *t* test. (**I**) Hydroxyproline levels were measured in the right lungs from control and cWT1^OE^ mice treated with bleomycin (*n* = 4–5/group). *P < 0.05, by 2-tailed Student’s *t* test. (**J**) Representative images of αSMA-stained lung sections from control and cWT1^OE^ mice treated with bleomycin. Scale bars: 100 μm. (**K**) Quantification of αSMA^+^ area in whole lung sections (*n* = 4–5/group). *P < 0.05, by 2-tailed Student’s *t* test. Tam, tamoxifen.

Studying pulmonary fibrosis using aged mice is crucial because aging is a significant risk factor for IPF, and aged mice better replicate the cellular and molecular environment of human IPF, providing a more accurate model for understanding disease mechanisms. Therefore, we overexpressed WT1 selectively in fibroblasts and assessed fibrotic changes in lungs using aged mice with or without bleomycin injury. As shown in Figure 8A, cWT1^OE^ and control mice (15 months old) were treated with 2 tamoxifen injections per week, and expression of survival and ECM gene expression were quantified on day 14. Overexpression of WT1 resulted in a significant increase in the expression of WT1 and antiapoptotic gene expression, including *Bcl2*, *Bcl3*, and *Bcl-XL* (Figure 8B). Further, western blot analysis of total lung lysates supported a significant increase in Col1α1, Bcl2, and Bcl-XL with overexpression of WT1 in cWT1^OE^ mice compared with control mice treated with tamoxifen (Figure 8, C and D). Masson’s trichrome staining exhibited modest or no significant increase in collagen staining in cWT1^OE^ mice compared with control mice following tamoxifen treatment (Supplemental Figure 13, A and B). To assess fibrotic responses with WT1 overexpression during bleomycin-induced injury, cWT1^OE^ and control mice (15 months old) were treated with bleomycin intratracheally and simultaneously with tamoxifen injections to overexpress WT1 (Figure 8E). Co-immunostaining of lung sections with antibodies against WT1 and vimentin revealed upregulation of WT1 in fibroblasts of cWT1^OE^ mice compared with control mice treated with bleomycin (Figure 8F). We assessed collagen staining in lung sections using Masson’s trichrome staining and observed a significant increase in collagen staining in cWT1^OE^ mice compared with control mice treated with bleomycin (Figure 8, G and H). Notably, we found that the total hydroxyproline levels were elevated in cWT1^OE^ mice compared with control mice treated with bleomycin (Figure 8I). Lung sections immunostained with antibodies against αSMA revealed a significant increase in αSMA staining area in the lungs of cWT1^OE^ mice compared with bleomycin-treated control mice (Figure 8, J and K), consistent with myofibroblast accumulation. Furthermore, Western blot analysis showed WT1 overexpression significantly increased the expression of the antiapoptotic protein Bcl2 and the ECM protein elastin in cWT1^OE^ mice compared with control mice treated with bleomycin (Supplemental Figure 14, A and B). Thus, our findings indicate that WT1 overexpression in fibroblasts of aged mice contributes to myofibroblast accumulation and collagen deposition during bleomycin-induced pulmonary fibrosis.

## Discussion

In this study, we used snRNA-Seq analyses to characterize matrix-embedded mesenchymal cell populations in the distal areas of IPF lungs. We identified 9 mesenchymal cell subpopulations, including WT1-expressing fibroblasts and myofibroblasts. Although it has been demonstrated that WT1 overexpression promotes fibroproliferation and ECM production, whether WT1 also impairs apoptotic clearance of lung fibroblasts resulting in progressive accumulation of myofibroblasts has not been established. Here, we demonstrate that expression of WT1, typically restricted to mesothelial cells during lung development, was aberrantly upregulated along with expression of antiapoptotic genes in distal lung fibroblasts in IPF samples. We conclude that the expression of WT1 in fibroblasts marked a pathogenic shift, contributing to the impaired clearance of fibroblasts and the progression of fibrotic remodeling in the lung.

The observed upregulation of WT1 in IPF fibroblasts suggests a unique and previously unrecognized role for this developmental transcription factor in lung fibrosis. Our in vitro and in vivo findings are consistent with this notion, as immunohistochemical analyses revealed that WT1 was predominantly localized in the nuclei of spindle-shaped cells within fibrotic lesions of the distal lung, and WT1 upregulation was associated with elevated fibroproliferation, ECM production, and resistance to apoptosis — all key processes in the development and maintenance of fibrotic lung lesions. The functional role of WT1 in fibroblast survival and ECM production was further supported by our loss- and gain-of-function studies, as WT1 knockdown in IPF fibroblasts resulted in a marked reduction in ECM-related gene expression and increased sensitivity to apoptosis. In vivo, overexpression of WT1 in fibroblasts exacerbated fibrosis in bleomycin-induced pulmonary fibrosis models, as evidenced by increased collagen deposition, myofibroblast accumulation, and impaired lung function. Furthermore, the deletion of WT1 in activated fibroblasts isolated from fibrotic lesions attenuated their proliferation and survival. These complementary in vitro and in vivo experiments establish WT1 as a pivotal regulator of fibroblast survival and fibrosis severity. WT1-expressing fibroblasts that accumulated in the distal lung regions expressed high levels of prosurvival and antiapoptotic BCL2 family genes. The high expression of WT1 in distal fibrotic lesions was consistent with the notion that WT1 may directly confer a cell survival advantage in addition to proliferative and matrix-producing functions. This hypothesis is also supported by the fact that WT1 is required to overcome apoptosis and potentiate proliferation in myeloid leukemia cells (26). Consistent with our data demonstrating that WT1 transcriptionally upregulated BCL2 expression, a study by Mayo et al. suggests that WT1 positively stimulates the BCL2 promoter through direct interaction (27). The antiapoptotic function of WT1 is not limited to fibroblasts in pulmonary fibrosis, as high WT1 expression in osteosarcoma has been shown to promote BCL2 levels and resistance to apoptosis (28). Similarly, a recent study suggests that WT1 may potentiate oncogenesis and tumor progression by transcriptionally upregulating BCL2 and that this mechanism may contribute to the heightened resistance to chemotherapy-induced apoptosis in diffuse anaplasia Wilms tumors (27).

Our study has several noteworthy limitations. The samples used in our analyses were collected exclusively from the lung periphery, which restricted our ability to investigate the involvement of mesenchymal cell types from other regions of the lung in the pathogenesis of pulmonary fibrosis. However, the use of stored tissue enabled us to process multiple samples simultaneously, minimizing batch effects. While we observed an accumulation of WT1-expressing fibroblasts in the distal regions of IPF lungs, our current methodology does not allow us to determine the extent of crosstalk between WT1^+^ cells and other fibroblast subpopulations in these regions, nor their distinct contributions to IPF pathogenesis. Supporting our observations, Habermann et al. identified HAS1^+^, WT1-expressing fibroblasts localized to subpleural regions, that were characterized by elevated expression of fibrosis-related collagen genes and activation of cellular stress and Th2 cytokine pathways (23).

In this study, we identified what we believe to be a new role for WT1 in promoting mesenchymal cell survival by regulating the expression of apoptosis-associated genes. This enhanced survival was accompanied by increased collagen deposition and accumulation of myofibroblasts in a bleomycin-induced model of pulmonary fibrosis in both young and aged mice. Notably, in the absence of injury, WT1 overexpression had minimal or no effect on the induction of survival genes or other profibrotic genes in the lungs of young mice but was sufficient to induce mild fibrosis in aged lungs. Given that IPF is a predominantly age-associated fibrotic lung disease, these findings suggest that age-related epigenetic alterations may have amplified the effects of WT1 in the aged lung. Indeed, previous studies have shown that aging is associated with altered chromatin accessibility, including increased histone acetylation, which could enhance the expression of fibrotic genes and contribute to persistent fibrosis (29). Together, these findings underscore the complex regulatory role of WT1 in fibroblast activation and highlight the need for future studies to dissect the mechanisms governing WT1-driven gene expression in aging tissues.

A study by Karki et al. suggests that WT1 loss in pleural mesothelial cells (PMCs) promotes their migration into the lung parenchyma and MMT. However, more recent studies have demonstrated that WT1 is upregulated in both mesothelial cells and (myo)fibroblasts in IPF, as well as in a mouse model of TGF-α–induced pulmonary fibrosis (18, 30). Consistent with these findings, our snRNA-Seq and immunostaining analyses revealed increased WT1^+^ mesothelial cells and (myo)fibroblasts in IPF. Both in vitro and in vivo studies using loss-of-function and gain-of-function models demonstrate that WT1 functions as a positive regulator of ECM gene expression, fibroproliferation, myofibroblast transformation, and pulmonary fibrosis (3). While the findings by Karki et al. provide important insights into mesothelial contributions to fibrosis, the broader body of evidence underscores a more complex role for WT1 in fibroblast activation and ECM remodeling. This refined understanding highlights WT1 as a potential therapeutic target in pulmonary fibrosis. Although transcription factors have traditionally been challenging to target therapeutically, strategies using small-molecule inhibitors and RNA-based approaches are emerging. In particular, several transcription factor–targeting therapies, including inhibitors of AP-1, JAK/STAT, NF-κB, and MYC, are currently under investigation, highlighting the potential feasibility of this approach (31–34). The selective upregulation of WT1 in fibrotic lesions, as opposed to control lung tissue, suggests that WT1 could serve as a promising therapeutic target with limited off-target effects in nonfibrotic tissues. This specificity contrasts with the widespread expression of prosurvival genes such as *BCL2* across multiple lung cell types, which could limit the therapeutic efficacy of BCL2 inhibitors like BH3 mimetics (e.g., ABT-262) due to their off-target effects (35, 36). Our findings thus provide a critical foundation for the development of targeted therapies that leverage the unique expression patterns of WT1 in IPF (3, 18). Indeed, WT1 has emerged as a potential therapeutic target in oncology because of its key role in cell growth and differentiation and limited expression in normal tissues (37, 38). WT1-specific immunotherapies, such as peptide vaccines, DC vaccines, and adoptive T cell therapies, are being designed to enhance the immune recognition and destruction of WT1-expressing cancer cells (37, 39). Small-molecule inhibitors targeting WT1 directly or its downstream signaling pathways are also being studied (9, 40). For example, WT1-specific antisense oligonucleotides have shown potential in preclinical models by reducing WT1 expression and impairing cancer cell growth (26). These approaches are also potentially promising for targeting fibroblasts in pulmonary fibrosis, although more research is needed to validate these strategies. Unlike broad TGF-β pathway inhibitors that affect multiple cell types and immune homeostasis, targeting WT1 may provide a more specific means to disrupt fibroblast activation and survival while minimizing off-target effects. Given its aberrant expression in pulmonary fibrosis, targeted delivery strategies, such as nanoparticle-based siRNA approaches, may help mitigate toxicity concerns (41, 42). Therefore, future studies are warranted to refine WT1-targeting approaches and assess their therapeutic efficacy in IPF and other fibrotic diseases.

In conclusion, our findings demonstrate that WT1 played a critical role in the pathogenic activation of fibroblasts in IPF. By promoting fibroblast proliferation, survival, and ECM production, WT1 contributed to the fibrotic remodeling that characterizes IPF. Targeting WT1 or its upstream regulators may offer a new therapeutic strategy for mitigating fibrosis and improving outcomes for patients with IPF. Further studies are warranted to explore the therapeutic potential of modulating WT1 expression or activity and to better understand the broader implications of WT1 reactivation in other fibrotic diseases.

## Methods

### Sex as a biological variable.

Our study included both male and female human samples, as well as male and female mice. Our findings were similar for both sexes, indicating that the reported results were not sex specific.

### Human samples.

Human lung tissue samples from patients with IPF and healthy controls were obtained through the Translational Pulmonary Science Center (TPSC) at the University of Cincinnati Medical Center. Lung tissues from individuals without documented lung disease were used as the control donor lungs. To safeguard patient anonymity, all samples and associated data were deidentified before being provided to the research team.

### Mouse strains.

The generation of *ROSA26:WT1-KTS*–knockin mice and *WT1^fl/fl^* mouse lines has been previously described (43, 44). To generate fibroblasts-specific *WT1^OE^* mice (*PDGFRa^CreERT^ WT1^OE^ cWT1^OE^*), WT1-knockin mice were crossed with *PDGFRα^CreERT^* mice (stock no. 018280, The Jackson Laboratory). WT1 overexpression was induced in adult cWT1^OE^ mice through Cre activation, which was achieved by administering tamoxifen twice weekly for 2 or 4 weeks, starting at 12–14 weeks (young mice) or 15 months (old mice) of age. The generation of doxycycline-inducible and Clara cell–specific TGF-α–overexpressing mice (*TGF-α^OE^* mice) has also been previously described (45). We bred *TGF-α^OE^* mice with *WT1^fl/fl^* mice to generate *TGF-α^OE^* *WT1^fl/fl^* mice. To induce TGF-α overexpression, mice were fed a diet containing doxycycline (62.5 mg/kg), leading to significant fibrotic lung disease within 4 weeks. Genotyping was performed using the primers listed in Supplemental Table 3, obtained from Invitrogen (Thermo Fisher Scientific) and Integrated DNA Technologies (IDT).

### Human lung sample collection and single nucleus isolation.

Frozen lung tissues were collected from the basal segments of the right lower lobe and carefully excised to obtain approximately 50 mg of tissue free of visible airway structures, vessels, mucin, or blood clots. Tissues were immediately placed into a nucleus isolation buffer (20 mM Tris-HCl pH 7.5, 292 mM NaCl, 2 mM CaCl2, 42 mM MgCl2, 1% CHAPS, and 2% BSA) and homogenized using a Dounce homogenizer to mechanically dissociate nuclei from cells. The resulting homogenate was filtered through a 40 μm strainer, and nuclei were pelleted by centrifugation at 300*g* for 2 minutes at 4°C. Nuclei were resuspended in 0.04% BSA buffer to a final concentration of 1,000 nuclei/μL. snRNA-Seq libraries were prepared using the 10X Genomics 5′ Kit v2, following the manufacturer’s protocol. Libraries were sequenced on an Illumina NextSeq 550 platform using 75 cycle paired-end sequencing.

### Data processing and QC.

Raw sequencing data were demultiplexed using Illumina’s bcl2fastq (version 2.20.0.422) software with default parameters, generating individual fastq files for each sample. These fastq files were aligned to the human reference genome (hg38) on 10X Genomics Cell Ranger, version 7.0.1, using 10X Genomics Cloud Analysis, which generated cell barcode, feature, and matrix files. Data were further processed in R Studio (version 2023.03.0) using the Seurat package (version 4.3.0) (46). Individual sample Seurat objects were created using.h5 files, in which Seurat objects went through QC scrutiny before final analysis. Low-quality cells were first filtered below the threshold of 1,000 detected genes and according to those with greater than 20% mitochondrial gene expression per cell (22). Later, doublets were identified and removed using DoubletFinder (version 2.0.3) (47), where artificial doublets were created on the basis of preliminary clustering, and cell clusters around the artificial clusters were classified as Doublets.

### snRNA-Seq analysis.

All analyses were performed using standard Seurat functions. The merged object was first log normalized to transform the gene expression values, whereby raw counts were normalized and scaled up by a factor of 1,000, followed by log transformation. Subsequently, 3,000 variable genes were identified for each individual object using the “Find Variable Features” function in Seurat. For these identified genes, data were scaled to have a zero mean across all cells and a variance of 1 using the “Scale Data” function. We used the “Run PCA” function and identified thirty principal components (PCs) and used them to identify nearest neighbors using the “Find Neighbors” function and clusters using the “Find Clusters” function. The resolution was adjusted to identify the predicted number of cell types. The reciprocal PCA protocol was adapted to integrate multiple Seurat objects and remove batch effects while preserving biological identity. Uniform manifold approximation and projection (UMAP) plots were used to visualize clustering with a resolution of 0.7 for all cells and 0.3 for major cell types using the “Run UMAP” function. For data analysis and visualizations using heatmaps and violin plots, data in the RNA slot were used.

### Cell-type annotation and differential gene expression analysis.

Seurat-generated cell clusters were categorized into 4 major cell populations on the basis of the canonical cell markers *COL1A2* for mesenchymal cells*, EPCAM* for epithelial cells, *PECAM1* for endothelial cells, and *PTPRC* for immune cells (Supplemental Figure 1). Each major type was further subdivided into subclusters following the same pipeline described above and was manually annotated using known cell-type markers (Supplemental Table 2). Multiplets, identified by the coexpression of markers from different cell types, were removed, and the final dataset was created by mapping all annotated subgroups (Figure 1C). To calculate the average gene expression level for each cell type per sample, the “Average Expression” function in Seurat was used, grouping cells either by sample alone or by both sample and cell type. Differentially expressed genes (DEGs) between cell types were identified using Seurat’s “Find All Markers” function with default parameters, which compared each cluster against all others to generate a gene list for each cell type. To assess differential expression between IPF and control lung cells, the “Find Markers” function was applied with default parameters. The Wilcoxon rank-sum test was used to generate *P* values for all DEG analyses. Gene set enrichment analysis was conducted using the “ToppFun” application of the ToppGene suite (48), and the results were visualized using Cytoscape (version 3.10.3) (49).

### Mouse model of bleomycin-induced pulmonary fibrosis.

Bleomycin was prepared by mixing sterile bleomycin sulfate powder (Teva Parenteral Medicines) with sterile normal saline. For the repetitive bleomycin injury model, 3 doses of bleomycin were administered intratracheally at 3-week intervals at a dose of 1.6 U/kg BW in a total volume of 50 μL sterile saline, and mice were euthanized at the end of the ninth week. For the single-dose bleomycin injury model, bleomycin was administered intratracheally at a dose of 3 U/kg BW in a total volume of 50 μL sterile saline, and mice were euthanized at the end of the third week.

To analyze lung function, mice were injected with 100 μL pentobarbital (65 mg/mL) intraperitoneally. A cannula was inserted into the trachea by puncturing it ventrally, and lung function parameters were measured using flexiVent (Scireq), following established protocols (50). For biochemical, transcriptomics, and pathologic analysis of lung tissues, animals were sacrificed, and the lungs were collected for histologic analysis, RNA, protein, and hydroxyproline.

### Hydroxyproline measurement.

Total lung hydroxyproline levels were quantified using a colorimetry-based method (catalog MAK008, MilliporeSigma) as described previously (51). Briefly, lung tissue was hydrolyzed with HCl and neutralized with sodium hydroxide. The neutralized hydroxyproline was oxidized to pyrrole followed by a reaction with Ehrlich’s reagent in perchloric acid, which forms a bright-colored chromophore, and measurement at a wavelength of 558 nm.

### Histology and IHC.

Histology and immunostaining were performed as described previously (52). In brief, the paraffin-embedded lungs were sectioned at a thickness of 5 μm and stained with Masson’s trichrome. For immunostaining, lung sections were deparaffinized, and antigen retrieval was performed with 10 mM citric acid (pH 6.0). The sections were then incubated with 5% donkey serum for 1 hour, followed by overnight incubation with species-specific primary antibodies. Next, the sections were incubated with species-specific secondary peroxidase antibodies and stained with DAB. Finally, the lung sections were mounted, and visualized with a Keyence BZ series microscope, and the area of staining was quantified with the BZ-X analyzer.

### Multiplex FISH.

Paraffin-embedded lung sections (5 μm thick) from control and IPF lung tissues were hybridized with *WT1* (catalog 415581-C1) and *ACTA2* (catalog 444771-C2) probes according to the manufacturer’s instructions (Advanced Cell Diagnostics, USA). In brief, lung sections were deparaffinized, pretreated with hydrogen peroxide, and treated with RNAscope Protease Plus (Advanced Cell Diagnostics, USA) for 15 minutes at room temperature (RT). Probes and RNAscope signal amplifiers were applied. Finally, DAPI-stained lung sections were mounted, visualized using a Leica STELLARIS 8 confocal microscope, and analyzed with Leica Microsystems LAS X image analysis software.

### Primary lung fibroblast cultures.

Adult human lung primary fibroblasts from control tissue, patients with IPF, and mice were isolated using collagenase digestion, as described previously (4), and then cultured in DMEM (10% FBS) for human fibroblasts or IMDM media for mouse fibroblasts (5% FBS). The culture medium was supplemented with glutamine, penicillin, streptomycin, amphotericin, and gentamycin. Lung-resident fibroblasts were isolated with negative selection using anti-CD45 microbeads, and the cells were passed through magnetic columns. The fibroblasts used in this study were from passages 1 to 3.

### Transfection and transduction of fibroblasts.

For stealth siRNA–mediated studies, IPF fibroblasts were transfected with control stealth siRNA (catalog 12935300, Invitrogen, Thermo Fisher Scientific) or WT1-specific siRNA (catalog HSS111388, Invitrogen, Thermo Fisher Scientific) for 72 hours using the Lipofectamine 3000 Transfection Kit (Life Technologies, Thermo Fisher Scientific) as described previously (10). Human primary fibroblasts were treated with a control adenovirus or WT1-overexpressing adenoviral particles as described previously (53). Cells were harvested after 48–72 hours for RNA or protein lysates, as mentioned previously (10). For Cre-mediated in vitro experiments, *TGF-a^OE^* *WT1^fl/fl^* mice were fed doxycycline food for 8 weeks, and the lungs were cultured for 5 days to isolate lung-resident fibroblasts as described above. The purified lung-resident fibroblasts were transduced with either Cre-expressing adenovirus (AdCre) or control adenoviral (Ad-Control) particles to excise loxP sites that flanked the *WT1* gene for 72 hours followed by a TUNEL assay, immunoblotting, and RNA isolation.

### Quantitative RT-PCR analysis.

Total RNA was extracted from lung tissues and primary cells using the RNeasy kit (Qiagen), with the concentration determined using a NanoDrop 2000 spectrophotometer (Thermo Fisher Scientific) as previously described (53). Reverse transcription was conducted using SuperScript III (Thermo Fisher Scientific), followed by real-time PCR using SYBR select master mix (Bio-Rad) and the CFX384 Touch Real-Time PCR instrument (Bio-Rad), with analysis performed using CFX Maestro software (version 4.0). Target gene transcripts from mouse samples were normalized to hypoxanthine-guanine phosphoribosyl transferase (Hprt), while human transcripts were normalized to human *ACTB*. RT-PCR primers for mice and humans used in this study (Invitrogen, Thermo Fisher Scientific and IDT) are provided in Supplemental Tables 4 and 5, respectively.

### Immunoblotting.

Immunoblot analysis was performed as described previously (54). Briefly, total lung tissue lysates and primary cell lysates were prepared using cell lysis buffer (Cell Signaling Technology, catalog 9803S) with A protease inhibitors cocktail (MilliporeSigma), and THE protein concentration was estimated using a BCA Protein Estimation kit (Thermo Fisher Scientific). After SDS-PAGE separation, proteins were transferred to a nitrocellulose membrane and blocked with 5% BSA (MilliporeSigma, catalog A9647) for 2 hours at RT and probed with specific primary antibodies in blocking buffer at 4°C overnight, followed by detection with HRP-linked secondary antibodies. Band intensities were quantified using Image Lab software 6.1 (Bio-Rad), with target proteins normalized to the internal control GAPDH. The primary and secondary antibodies used in this study are listed with their dilutions in Supplemental Table 6.

### Measurement of collagen in fibroblast-conditioned media.

IPF fibroblasts were transfected with either control siRNA or WT1-specific siRNA and cultured for 72 hours in DMEM supplemented with 0.5% FBS. Following 72 hours of knockdown, conditioned media were collected and processed as previously described (55). Briefly, media samples were passed through a 0.45 μm filter to remove debris and subsequently concentrated using Amicon Ultra Centrifugal Filters (Thermo Fisher Scientific). The concentrated media were then mixed with 1× SDS loading buffer, denatured at 98°C for 5 minutes, and subjected to Western blot analysis to detect collagen.

### BrdU cell proliferation assay.

Cell proliferation was evaluated using the BrdU kit (Cell Signaling Technology) as described previously (3). Briefly, lung-resident fibroblasts isolated from control and cWT1^OE^ mice were cultured for 48 hours under low-serum conditions (0.5% serum), with BrdU labeling solution added after 24 hours in culture. Following 24 hours of BrdU labeling, cells were fixed, and BrdU immunodetection was performed according to the manufacturer’s instructions.

### TUNEL assay.

Human or mouse fibroblasts were treated with siRNA using the Lipofectamine 3000 transfection kit or transduced with adenovirus for 48 hours as described previously (53), followed by treatment with species-specific anti-Fas antibody (250 ng/mL, 05-201, clone CH11, MilliporeSigma) for 24 hours. Stealth negative control siRNA (catalog 12935300, Invitrogen, Thermo Fisher Scientific) and human WT1 stealth siRNA (catalog HSS111388, Invitrogen, Thermo Fisher Scientific) were purchased from Invitrogen (Thermo Fisher Scientific). Cells were then fixed with 4% paraformaldehyde, and nuclei were stained using DAPI. Assessment of DNA fragmentation in apoptotic cells was conducted using the TUNEL method with the In Situ Cell Death Detection kit, TMR red (Roche Diagnostics), following the manufacturer’s instructions. Imaging was performed at an original magnification of ×20 using a Nikon AIR-A1 confocal microscope, and quantification was carried out using MetaMorph imaging software (version 6.2, Molecular Devices).

### Immunofluorescence and confocal imaging.

Immunofluorescence and confocal imaging were performed as previously described (9). Briefly, mouse lung tissues from control and cWT1^OE^ mice were fixed in 1:10 diluted formalin, embedded in paraffin, and sectioned at 5 μm thickness. Sections were deparaffinized followed by citric acid (pH 6.0) antigen retrieval, blocked using 5% normal donkey serum, and then incubated with primary antibodies overnight at 4°C. The following day, the samples were incubated with species-specific secondary anti–donkey Alexa Fluor 488 and 594 antibodies, and nuclei were stained with DAPI. Images were captured using a Leica STELLARIS 8 confocal microscope and analyzed using Leica Microsystems LAS X image analysis software. Antibody details and dilutions are provided in Supplemental Table 5.

### Statistics.

All data were analyzed using GraphPad Prism, version 10.2.3 for Windows (GraphPad Software). For multiple comparisons, 1-way ANOVA with Tukey’s test was performed. Statistical significance between 2 groups was determined using Student’s 2-tailed *t* test. Data a presented as the mean ± SEM to indicate variability. *P* values of less than 0.05 were considered statistically significant.

### Human and animal study approval.

Human lung sample collection and usage procedures were conducted in compliance with ethics guidelines and approved by the University of Cincinnati IRB (no. 2013-8157), ensuring the protection of study participants and adherence to confidentiality protocols. Mice were housed under specific pathogen–free conditions at the University of Cincinnati, an American Association for the Accreditation of Laboratory Animal Care–accredited (AAALAC-accredited) facility. All animal experiments were conducted following protocols approved by the University of Cincinnati IACUC.

### Data availability.

Raw and processed sequencing data have been uploaded to the NCBI’s Gene Expression Omnibus (GEO) database (GEO GSE279404). All data point values presented in the graphs are included in the Supporting Data Values file.

## Author contributions

HHE, VS, and SKM devised the project, were involved in designing and executing the experiments, analyzed data, and wrote the manuscript. PKP performed immunostaining. CPV, AGJ, HM, DP, and MBJ performed bioinformatics analysis and edited the manuscript. SKH, FXM, and NG provided human lung tissue samples and edited the manuscript. CE and AS provided the WT1-transgenic mice and edited the manuscript.

## Supplementary Material

Supplemental data

Unedited blot and gel images

Supplemental video 1

Supplemental video 2

Supplemental video 3

Supporting data values

## Version 1 06/10/2025

In-Press Preview

## Version 2 08/01/2025

Electronic publication
