# Supplementary material for: Wilms tumor 1 impairs apoptotic clearance of fibroblasts in distal fibrotic lung lesions
Source: J Clin Invest. 2025 Jun 10;135(15):e188819. doi: 10.1172/JCI188819 (PMC12321392; doi:10.1172/JCI188819)
Supplement: Supplemental data [file jci-135-188819-s088.pdf]

Wilms' tumor 1 impairs apoptotic clearance of fibroblasts in distal fibrotic lung lesions

Harshavardhana H. Ediga[1], Chanukya P. Vemulapalli[1], Vishwaraj Sontake[2], Pradeep K. Patel[1], Hikaru Miyazaki[2], Dimitry Popov[2], Martin B Jensen[2], Anil G. Jegga[3,4], Steve K. Huang[5], Christoph Englert[6], Andreas Schedl[7], Nishant Gupta[1], Francis X. McCormack[1], and Satish K. Madala[1]*

[1] Division of Pulmonary, Critical Care and Sleep Medicine, Department of Internal Medicine, University of Cincinnati, Cincinnati, OH, USA.

[2] Gordian Biotechnology, South San Francisco, CA, USA.

[3] Division of Biomedical Informatics, Cincinnati Children's Hospital Medical Center, Cincinnati, OH, USA,

[4] Department of Pediatrics, University of Cincinnati College of Medicine, Cincinnati, OH, USA.

[5] Division of Pulmonary and Critical Care Medicine, University of Michigan Medical School, Ann Arbor, MI, USA.

[6] Molecular Genetics Lab, Leibniz Institute on Aging – Fritz Lipmann Institute, Jena, Germany.

[7] Institute of Biology Valrose, University of Sophia-Antipolis, Nice, France.

*** Correspondence:** Satish K. Madala, Division of Pulmonary, Critical Care and Sleep Medicine, The University of Cincinnati, CVC4938, 231 Albert Sabin Way, Cincinnati, OH 45267. E-mail: madalash@ucmail.uc.edu

Supplementary Figure 1

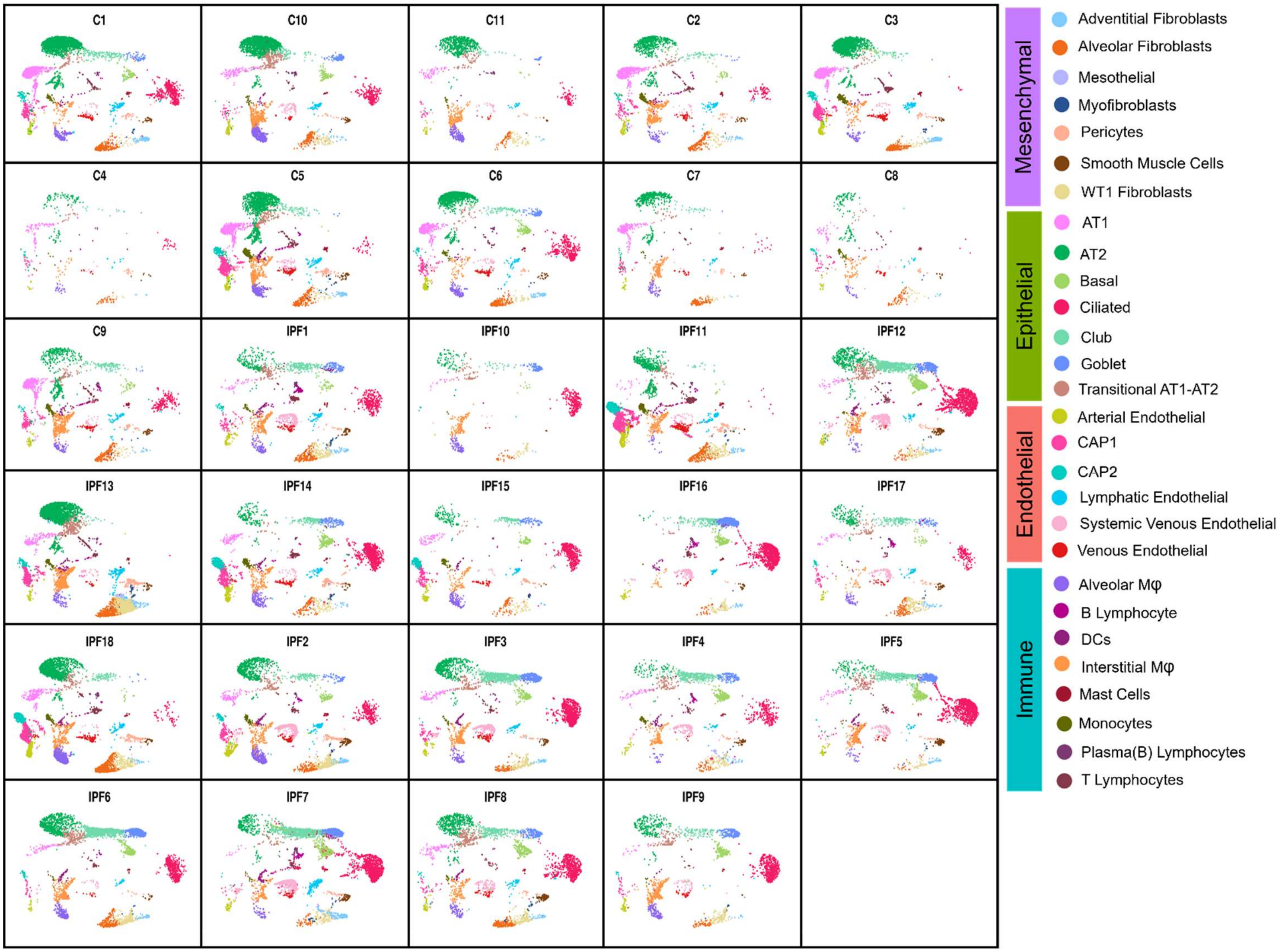

Supplementary Figure 1. The plots show UMAP layouts for all cell types from the control (N = 11, C1 to 11) and IPF (N = 18; IPF1 to IPF18) lung samples. Plots are colored by cell identity annotations.

Supplementary Figure 2

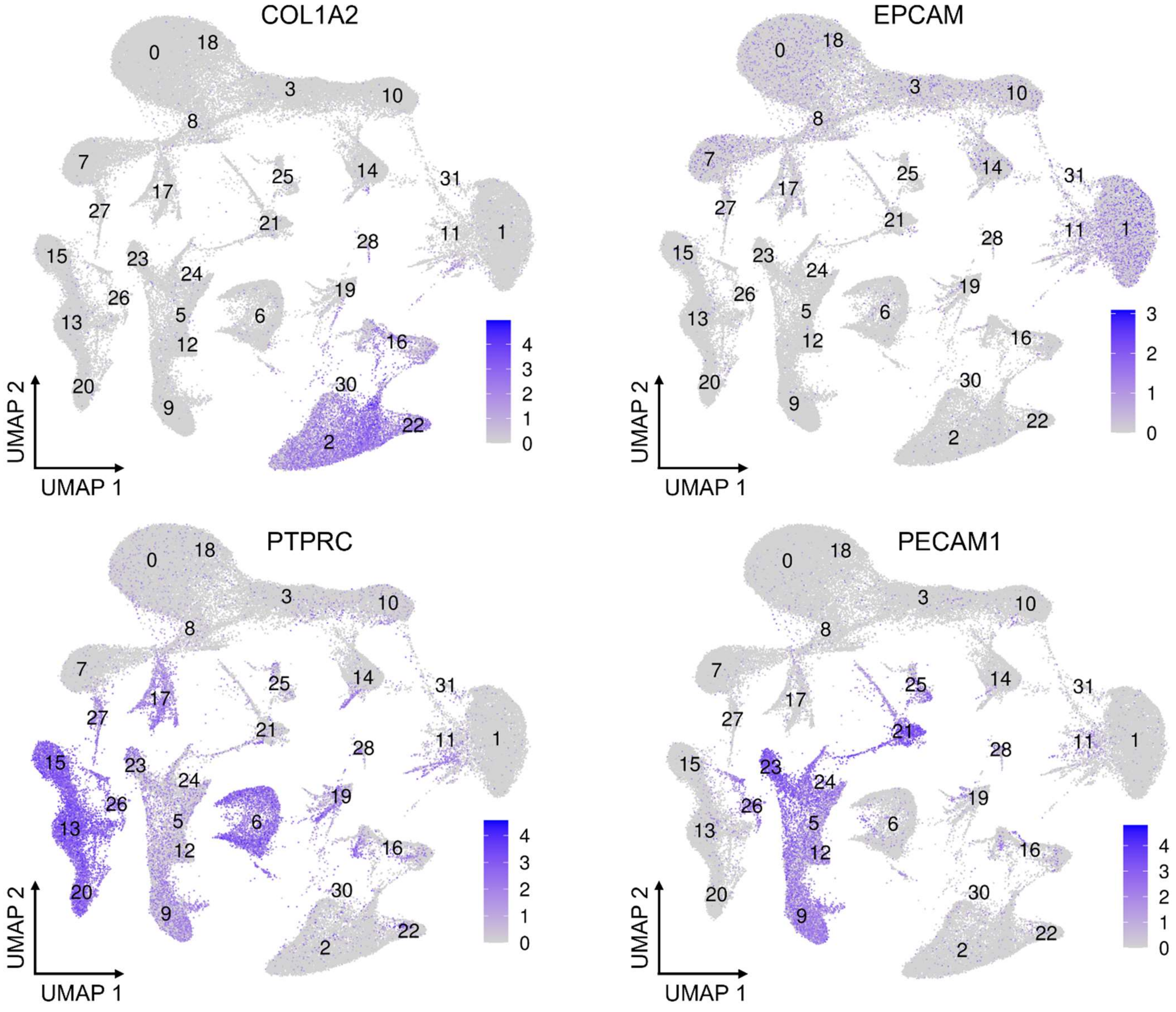

Supplementary Figure 2. UMAP plots show the normalized gene expression of COL1A2, EPCAM, PTPRC, and PECAM1 of all cells for identifying mesenchymal, epithelial, immune, and endothelial cell populations, respectively. Color intensity reflects expression levels, with darker shades indicating higher expression.

Supplementary Figure 3

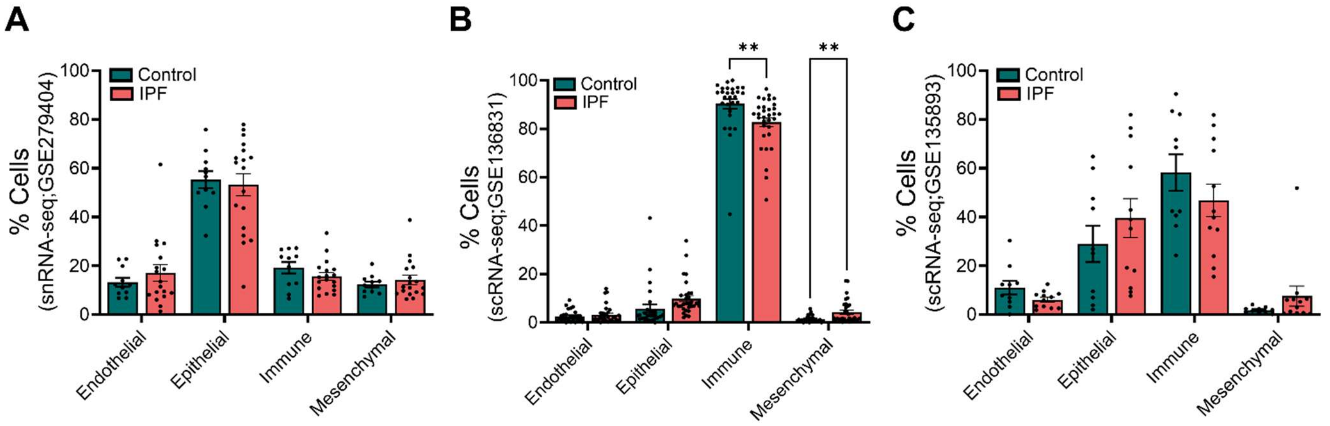

Supplementary Figure 3. The percent distribution of all four major cell categories as a proportion of all sampled cells in control and IPF lungs across different single-cell datasets, including **(A)** snRNA-seq (GSE279404), **(B)** scRNA-seq (GSE136831), and **(C)** scRNA-seq (GSE135893)**.** Each dot represents a single subject, and multiple t-test was used for comparisons between control and IPF (** $p < 0.01$).

Supplementary Figure 4

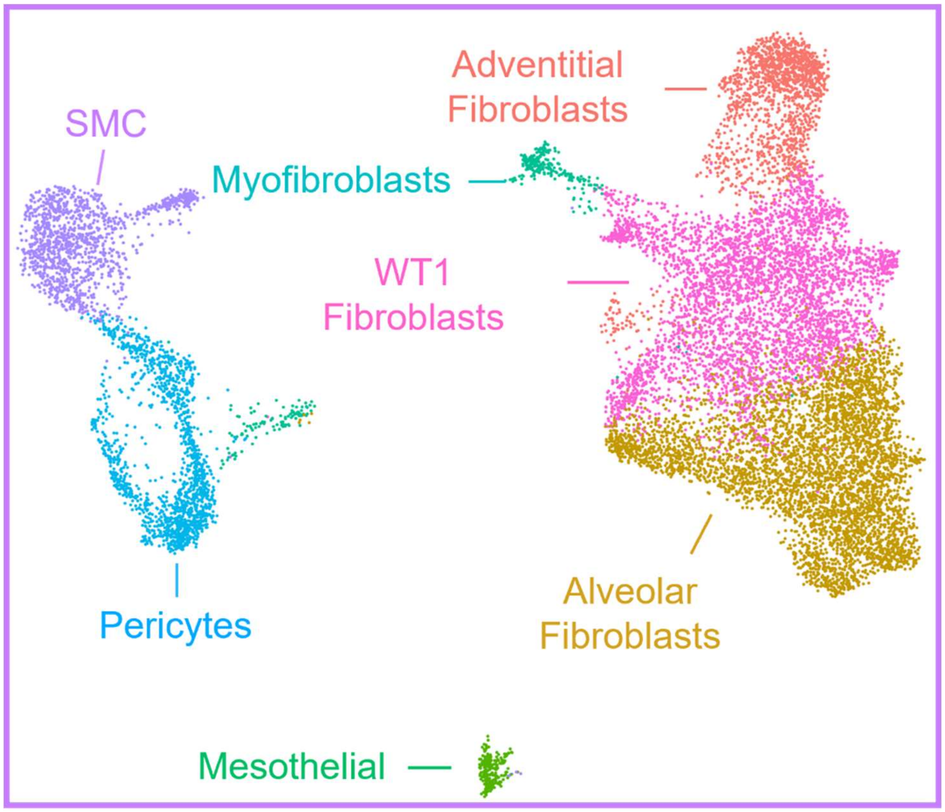

Supplementary Figure 4. UMAP plot shows color-coded 7 unique mesenchymal cell populations identified after the sub-clustering of all mesenchymal cells.

Supplementary Figure 5

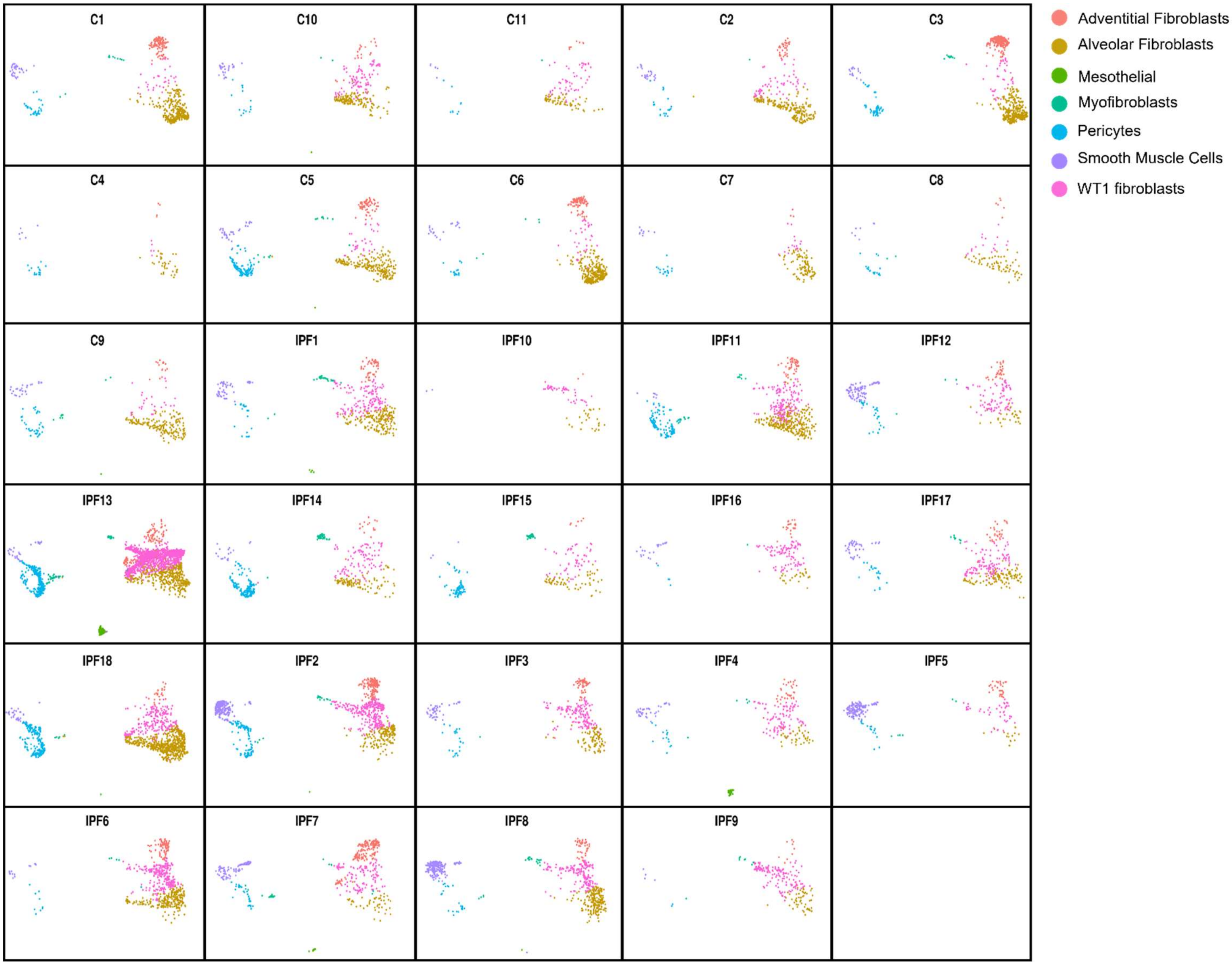

Supplementary Figure 5. The plots show UMAP layouts for mesenchymal cell populations from the control (N = 11, C1 to 11) and IPF (N = 18; IPF1 to IPF18) lung samples. Plots are colored by cell identity annotations.

Supplementary Figure 6

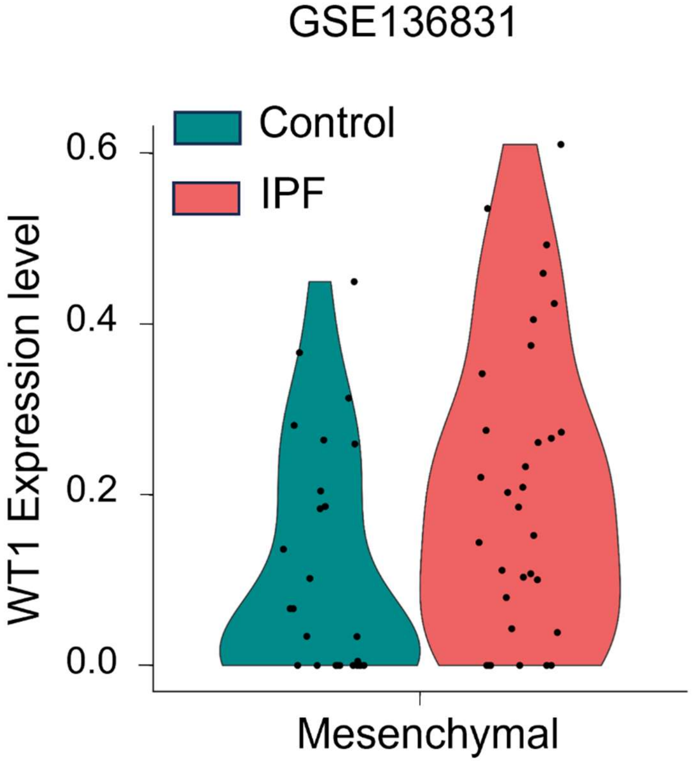

Supplementary Figure 6. Violin plot showing the average WT1 expression in the mesenchymal population comparing control and IPF subjects in a publicly available dataset (GSE136831). Each dot represents an individual subject. The p-value was calculated using the Wilcoxon rank-sum test (* $p < 0.05$).

Supplementary Figure 7

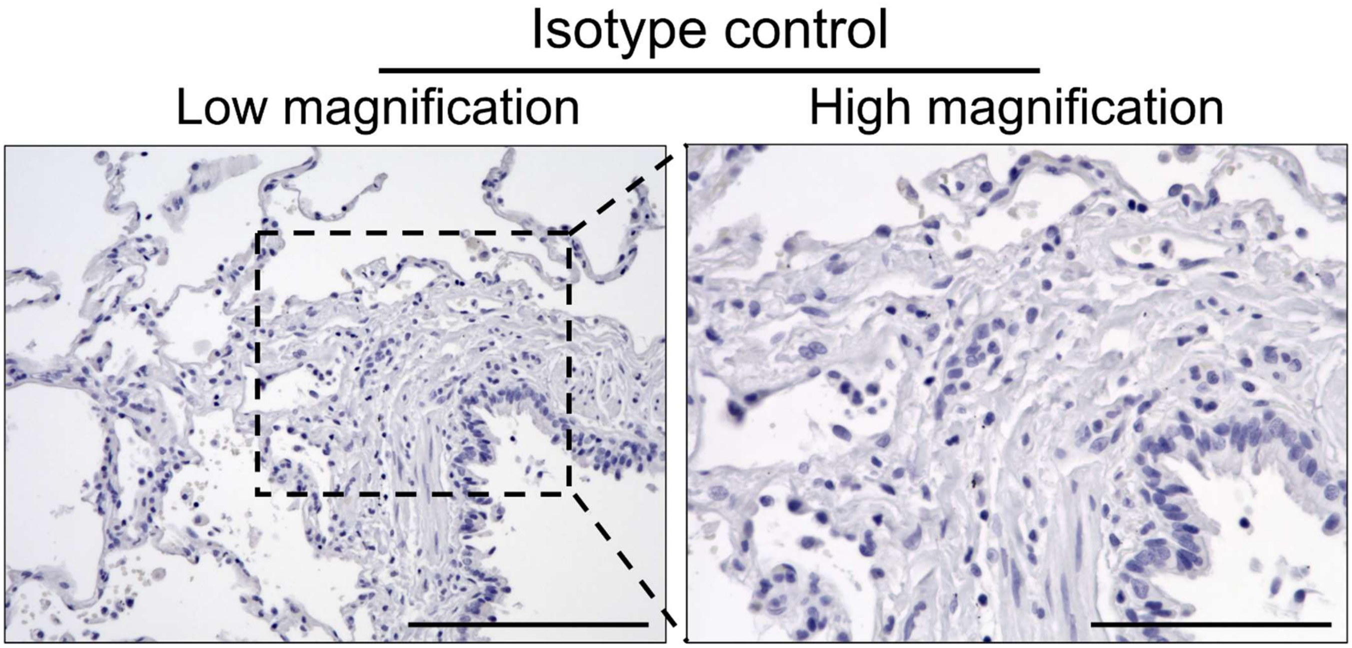

Supplementary Figure 7. Representative images of lung sections immunostained with an isotype control antibody (Rabbit IgG). Images were captured at low (20X; Scale bar, 200 μm) and high (40X; Scale bar, 100 μm).

Supplementary Figure 8

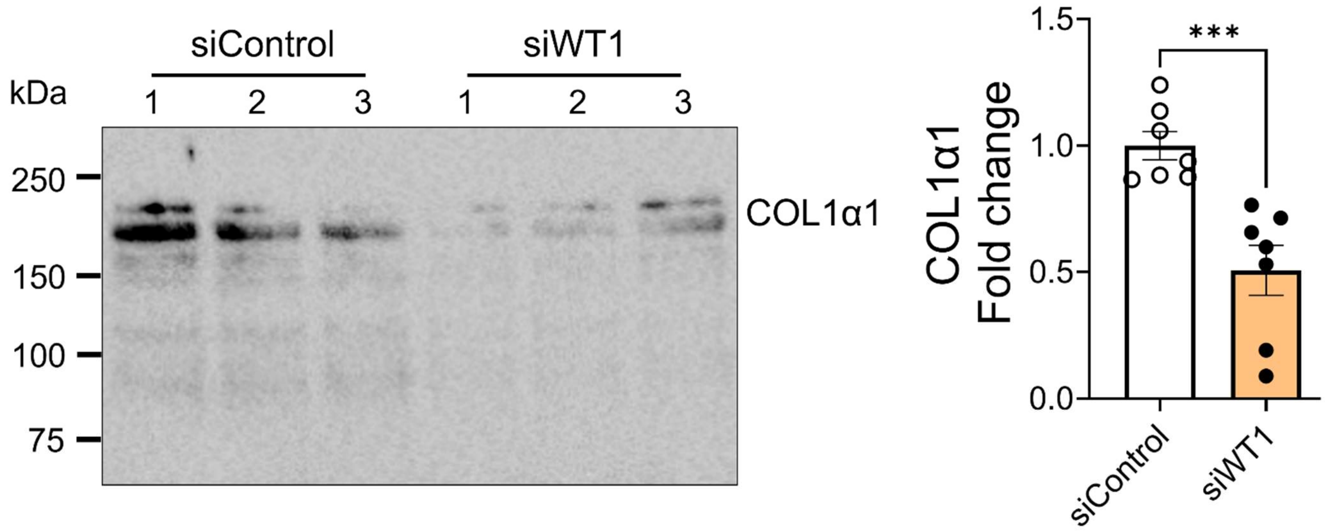

Supplementary Figure 8. The loss of WT1 attenuates collagen secretion in IPF fibroblasts. Conditioned media were collected from IPF fibroblasts treated with either control or WT1-specific siRNA for 72 hr and immunoblotted with an antibody against COL1α1. COL1α1 protein levels in conditioned media were shown as fold change normalized to total cells using a bar graph. Student's two-tailed *t*-test was used (** $p < 0.001$; n=7/group). Data is the cumulative result of two independent experiments with similar findings.

Supplementary Figure 9

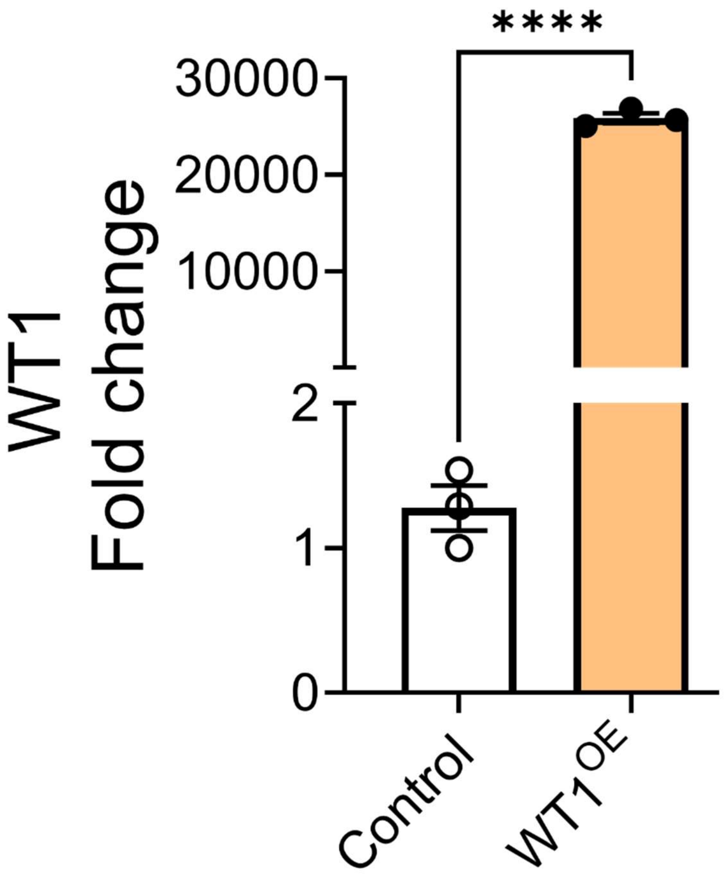

Supplementary Figure 9. Overexpression of WT1 using Adenovirus in normal lung fibroblasts. Quantification of *WT1* transcripts using RT-PCR in normal lung resident fibroblasts transduced with either control or WT1-Adeno viral particles for 72 hr. Student's two-tailed *t*-test was used (**** $p < 0.0001$; n=3/group).

Supplementary Figure 10

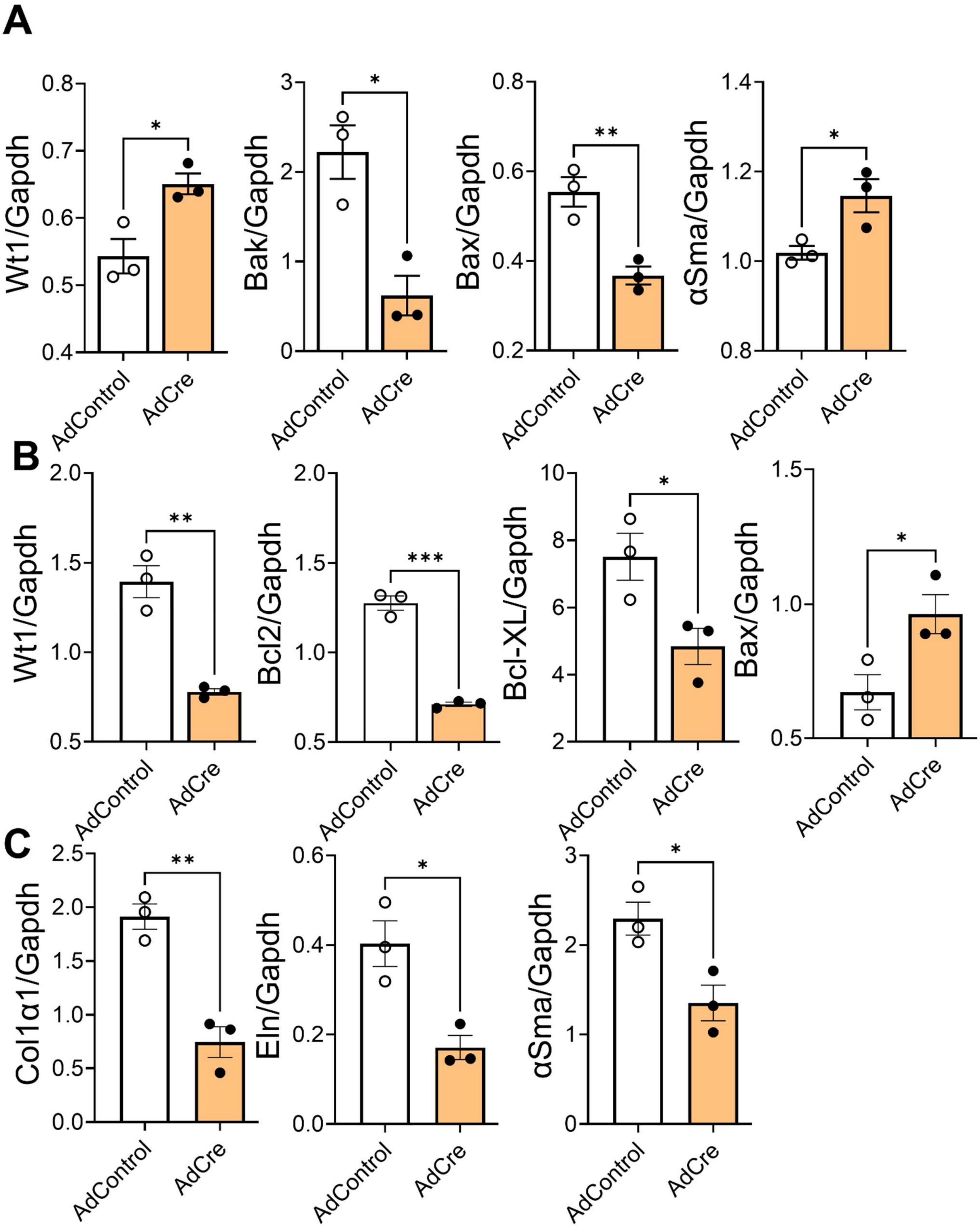

Supplementary Figure 10. (A) Quantification of Wt1, Bak, Bax, and αSma protein levels were normalized to Gapdh. Student's two-tailed t-test was used (*$p < 0.05$, **$p < 0.01$; n=3/group). (B) Quantification of Wt1, Bcl2, Bcl-XL, Bax, Eln, and αSma protein levels normalized to Gapdh. Student's two-tailed t-test was used (*$p < 0.05$, ** $p < 0.01$, *** $p < 0.001$; n=3/group). (C) Quantification of Col1α, Fn1, Eln, and αSma protein levels normalized to Gapdh. Student's two-tailed t-test was used (*$p < 0.05$, ** $p < 0.01$; n=3/group).

Supplementary Figure 11

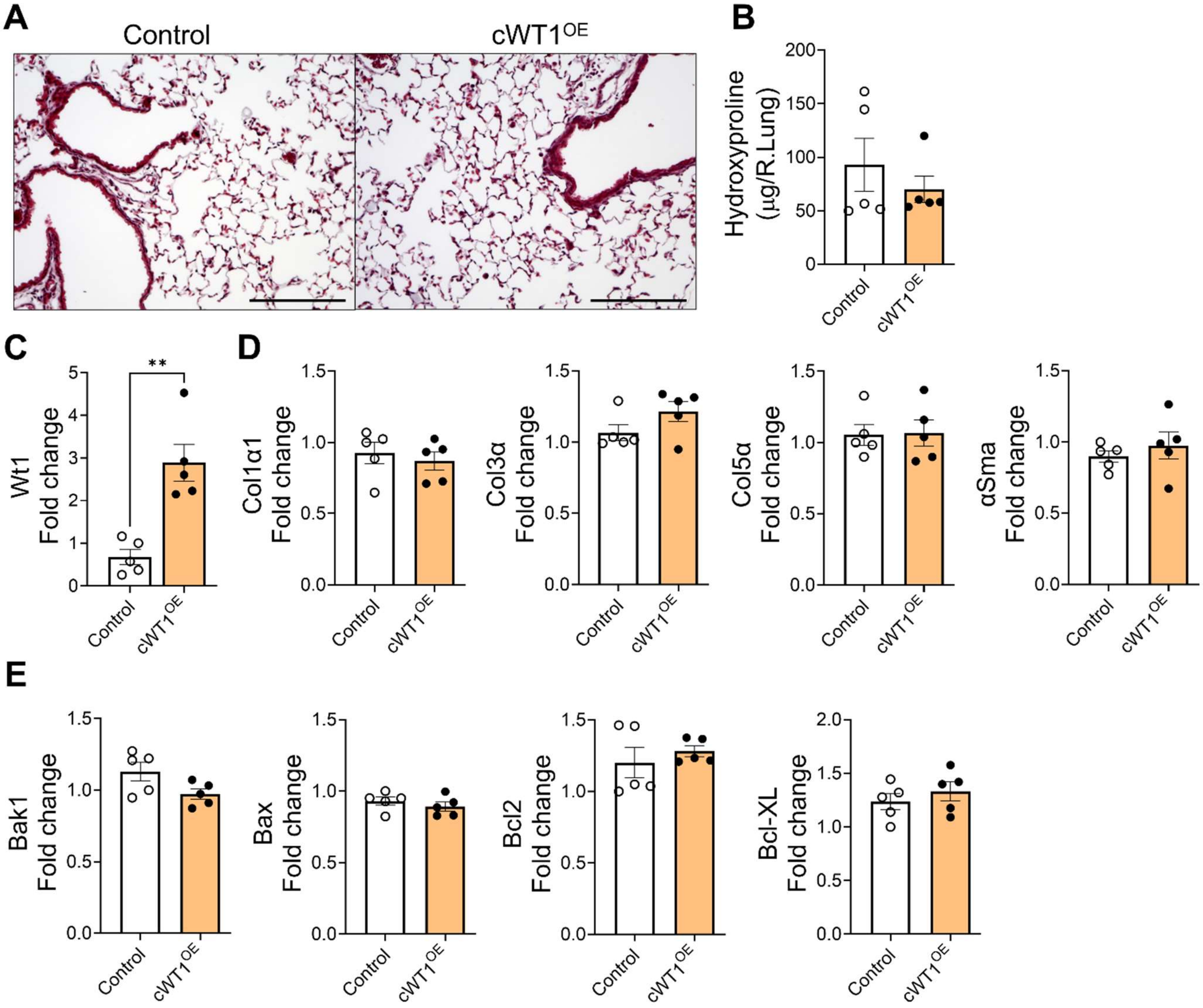

Supplementary Figure 11. (A) Masson's trichrome-stained lung sections from young age control and *cWT1*^{OE} mice treated with tamoxifen via intraperitoneal injection for two weeks. Images were captured at 20X original magnification. Scale bar, 200 μm. (B) Hydroxyproline levels were measured in the right lungs of control and cWT1^{OE} mice treated with tamoxifen via intraperitoneal injection for two weeks (n=5/group). (C) Quantification of WT1 gene transcripts by RT-PCR in the total lung RNA isolated from control and *cWT1*^{OE} mice treated with tamoxifen via intraperitoneal injection for two weeks. Student's two-tailed *t*-test was used (**$p < 0.01$; n=5/group). (D-E) Quantification of ECM (*Col1α1, Col3α*, and *Col5α*) and apoptosis-associated gene transcripts (*Bak1, Bax, Bcl2,* and *Bcl-XL*) from control and cWT1^{OE} mice treated with tamoxifen via intraperitoneal injection for two weeks.

Supplementary Figure 12

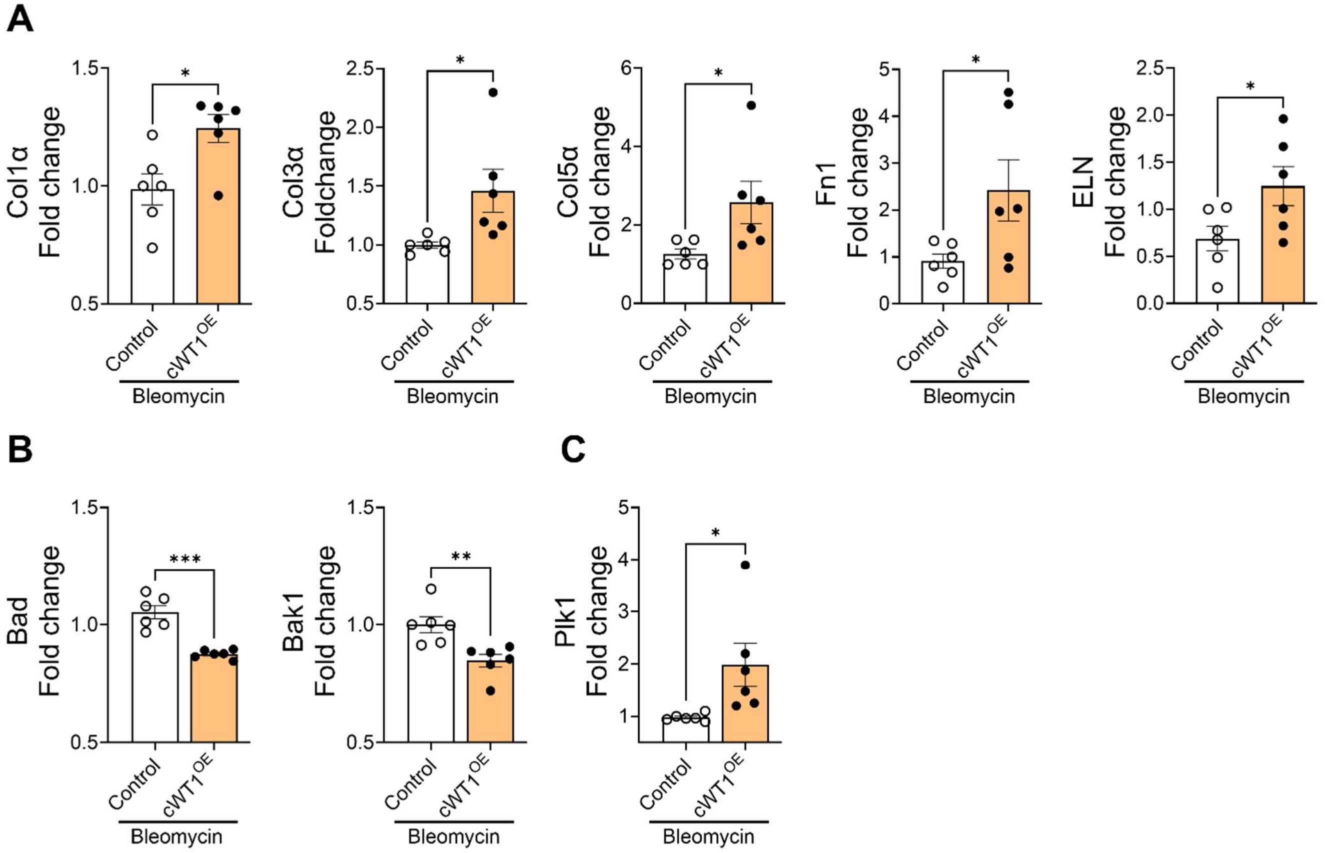

Supplementary Figure 12. (A) Quantification of ECM gene transcripts (*Col1α1, Col3α, Col5α, Eln,* and *Fn1*) from control and *cWT1OE* mice treated with bleomycin. Multiple unpaired t-test was used (* $p < 0.05$; n= 6/group). (B) Quantification of pro-apoptotic (*Bad* and *Bak*) gene transcripts from control and *cWT1OE* mice treated with bleomycin. Student's two-tailed t-test was used (***$p < 0.001$; **$p < 0.01$; n=6/group). (C) Quantification of *Plk1* gene transcripts from control and cWT1OE mice treated with bleomycin. Student's two-tailed t-test was used (*$p < 0.05$; n=6/group).

Supplementary Figure 13

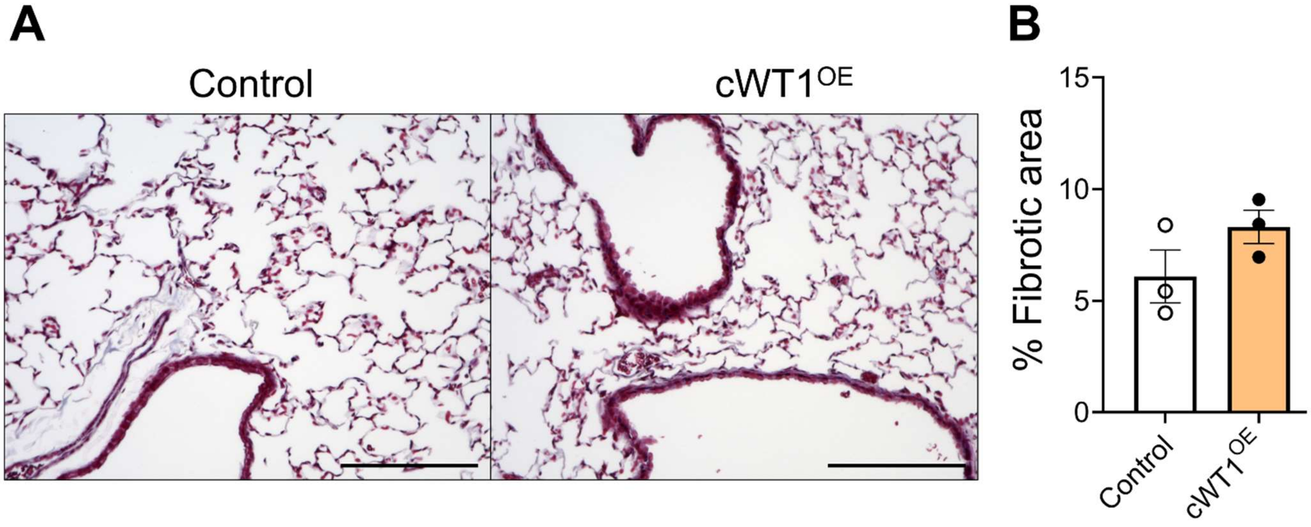

Supplementary Figure 13. (A) Masson's trichrome stained lung sections from old age control and *cWT1^OE^* mice treated with tamoxifen via intraperitoneal injection for two weeks. Images were captured at 20X original magnification. Scale bar, 200 µm. (B) Percent fibrotic area was quantified in control and cWT1^{OE} mice using BZ-X image analysis. (n=3/group).

Supplementary Figure 14

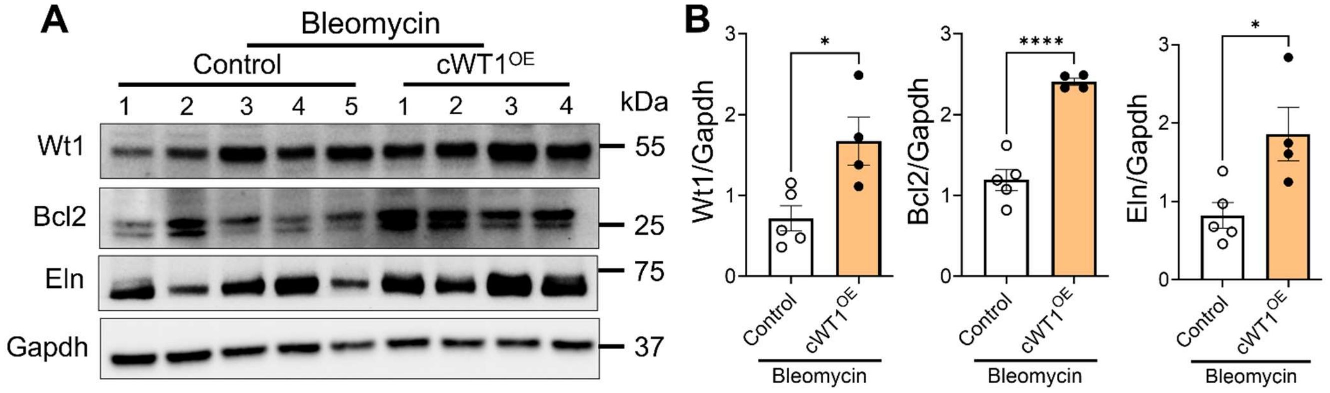

Supplementary Figure 14. WT1 increases the molecular markers of survival and ECM during bleomycin-induced pulmonary fibrosis. (A) The total lung lysates were immunoblotted with antibodies against Wt1, Bcl2, Eln, and Gapdh from old age control and *cWT1*^{OE} mice treated with bleomycin. (B) Wt1, Bcl2, and Eln protein levels were normalized to Gapdh and shown as fold change using a bar graph. Student's two-tailed *t*-test was used (* $p < 0.05$; **** $p < 0.0001$; n=4-5/group).

Supplementary Table S1. Clinical characteristics and total number of nuclei isolated from the distal lung regions of individuals with IPF and normal donors.

Condition	Samples(n)	Age	Sex (M/F)	Number of nuclei
IPF	18	62.3 ± 19.3	13/5	76992
Control	11	53 ± 6.6	6/5	40088

Supplementary Table S2. Markers used for cell annotation in snRNA-seq analysis.

Population	Cell Type Annotation	Marker(s)
Mesenchymal	Alveolar Fibroblasts	MYLK, TCF21, ADGRB3, DCC, LIMCH1
Mesenchymal	Adventitial Fibroblasts	MFAP5, SCARA5, IGF1, SPOCK1
Mesenchymal	Myofibroblasts	DACH2, ANO4, ITGBL1
Mesenchymal	WT1 fibroblasts	WT1, LUM, COL1A1, POSTN, COL3A1
Mesenchymal	Mesothelial	WT1, MSLN, CA12, MIR31HG, RBFOX1
Mesenchymal	SMCs	MYH11, ACTA2, LGR6, LDB3
Mesenchymal	Pericytes	LAMC3, TRPC6, PDGFRB, DGKG
Endothelial	Arterial Endothelial	GJA5, DKK2, ENPP2, ARL15
Endothelial	Lymphatic Endothelial	PROX1, MMRN1, CCL21, RELN, DLGAP2
Endothelial	Venous Endothelial	ACKR1, SULT1E1, HDAC9, ADGRG6, FAM155A
Endothelial	Systemic Venous Endothelial	COL15A1, VWA1, ESR2, MYRIP, POSTN
Endothelial	CAP1	APLNR, GPIHBP1, IL7R, FCN3, NRXN3
Endothelial	CAP2	CAR4, APLN, EDNRB, HPGD, TBX2
Epithelium	AT1	AGER, RTKN2, NCKAP5, EMP2, SCEL
Epithelium	AT2	SFTPC, LAMP3, LRRK2, ABCA3, SFTPB
Epithelium	Basal	KRT5, TP63, MIR205HG, KRT15, EYA2
Epithelium	Ciliated	FOXJ1, RSPH1, PTPRT, DCDC1, DNAH12
Epithelium	Club	SCGB3A2, HMCN1, SLC4A5
Epithelium	Goblet	MUC5AC, SPDEF, MUC5B, SCGB1A1, SCGB3A1
Epithelium	Translational AT1-AT2	Express both AT1 (AGER, RTKN2, NCKAP5, EMP2, SCEL) and AT2 markers (SFTPC, LAMP3, LRRK2, ABCA3, SFTPB)
Immune	Alveolar Macrophages	MSR1, MCEMP1, PPARG, LSAMP
Immune	Interstitial Macrophages	F13A1, STAB1, FMN1, ABCA1
Immune	Monocytes	VCAN, S100A8, LILRB2, LILRA5
Immune	B Lymphocytes	MS4A1, CD19, BLK, BACH2
Immune	Plasma (B) Lymphocytes	FCRL5, JCHAIN, TXNDC5, IGKC
Immune	T Lymphocytes	CD3E, IL7R, ITK, CD247
Immune	DCs	HDAC9, DC1, FCGR2B, CLEC10A, PALD1
Immune	Mast Cells	CPA3, TPSD1, GATA2, MS4A2

Supplementary Table S3. The list of mouse genotyping primers used in the study.

Gene Symbol	Forward primer	Reverse primer
CC10	ACTGCCCATTGCCCAAACAC	AAAATCTTGCCAGCTTTCCCC
TetO	CCTGTTCGCTCTGGGTATTGTGTT	CGTGGTCCGCTGATTTCTTCTCTA
PDGFRα CreERT	TCA GCC TTA AGC TGG GAC AT	ATG TTT AGC TGG CCC AAA TG
$WT1^{fl/fl}$	GCC CTA CAG CAG GTA AGA AGG	CTG GAG ACC TGA GAC AAG CA
$WT1^{OE}$	CACCAAAGGAGACACACAGGT	GGGCTTTTCACCTGTATGAG

Supplementary Table S4. The list of mouse RT-PCR primers used in the study.

Gene Symbol	Forward primer	Reverse primer
Acta2	TGACGCTGAAGTATCCGATAGA	CGAAGCTCGTTATAGAAAGAGTGG
Bad	GGAGCAACATTCATCAGCAG	TACGAACTGTGGCGACTCC
Bak1	GGAATGCCTACGAACTCTTCA	CCAGCTGATGCCACTCTTAAA
Col1a1	CATGTTCAGCTTTGTGGACCT	GCAGCTGACTTCAGGGATGT
Col1a2	AAGGGTGCTACTGGACTCCC	TTGTTACCGGATTCTCCTTTGG
Col3a1	TCCCCTGGAATCTGTGAATC	TGAGTCGAATTGGGGAGAAT
Col5a	CTACATCCGTGCCCTGGT	CCAGCACCGTCTTCTGGTAG
Eln	TGGAGCAGGACTTGGAGGT	CCTCCAGCACCATACTTAGCA
Fn1	CGGAGAGAGTGCCCCTACTA	CGATATTGGTGAATCGCAGA
Hprt	GCCCTTGACTATAATGAGTACTTCAGG	TTCAACTTGCGCTCATCTTAGG
Plk1	TTGTAGTTTTGGAGCTCTGTCG	CAGTGCCTTCCTCCTCTTGT
Wt1	CAG ATG AAC CTA GGA GCT ACC TTA AA	TGC CCT TCT GTC CAT TTC A

Supplementary Table S5. The list of human RT-PCR primers used in the study.

Gene Symbol	Forward primer	Reverse primer
ACTA2	GCTTTCAGCTTCCCTGAACA	GGAGCTGCTTCACAGGATTC
β-ACTIN	CCAACCGCGAGAAGATGA	CCAGAGGCGTACAGGGATAG
BAX	AGCAAACTGGTGCTCAAGG	CTTGGATCCAGCCCAACA
BIM	ACTGGAGAGCTCATTGCAGAC	AAATACCAGGACCCGAAGGT
BCL2	AGTACCTGAACCGGCACCT	GCCGTACAGTTCCACAAAGG
BCL2-L2	TGGATGGTGGCCTACCTG	CGTCCCCGTATAGAGCTGTG
BCL-XL	GCCACTTACCTGAATGACCAC	TGCTGCATTGTTCCCATAGA
BCL3	GCCTCAGCTCCAATGGTC	GAGGAGCCATGGGGAATC
COL1A1	GGGATTCCCTGGACCTAAAG	GGAACACCTCGCTCTCCA
COL6A3	ATGAGGAAACATCGGCACTTG	GGGCATGAGTTGTAGGAAAGC
COL16A1	CCACCAGAAGACGTGGTATCT	CAGGACACAAAGTCGCCATC
HSP90B1	GCTGACGATGAAGTTGATGTGG	CATCCGTCCTTGATCCTTCTCTA
PIM1	GGCTCGGTCTACTCAGGCA	GGAAATCCGGTCCTTCTCCAC
WT1	AGCTGTCCCACTTACAGATGC	CCTTGAAGTCACACTGGTATGG

Supplementary Table S6. The list of antibodies and their dilutions used in the study.

Antibody	Dilution			# Catalog, Clone	Company
	IHC	IF	WB		
ACTA2	1:20000	1:2000	1:20000	A5228 (1A4)	Sigma
BCL-XL			1:1000	2764S (54H6)	CST
BCL2			1:1000	3498S (D17C4)	CST
BAD			1:1000	9292S	CST
BAK			1:1000	12105S (D4E4)	CST
BAX			1:1000	2772S	CST
COL1α1			1:1000	91144S (E8I9Z)	CST
COL1α1			1:500	Sc-293182 (3G3)	Santa Cruz Biotechnology
COL1α1			1:1000	1310-01	Southern Biotech
ELN			1:500	15257-1-AP	Proteintech
FN1			1:500	Sc-9068	Santa Cruz Biotechnology
FAS			1:1000	8023S (4C3)	CST
GAPDH			1:2000	A300-641	Bethyl Laboratories
Vimentin		1:50		Sc-7557	Santa Cruz Biotechnology
WT1	1:200	1:50		Ab89901 (CAN-R9-(IHC)-56-2)	Abcam

WT1			1:500	12609-1-AP	Proteintech
