# Supplementary material for: Wilms tumor 1 impairs apoptotic clearance of fibroblasts in distal fibrotic lung lesions
Source: J Clin Invest. 2025 Jun 10;135(15):e188819. doi: 10.1172/JCI188819 (PMC12321392; doi:10.1172/JCI188819)

Full unedited blots for Figure 3E

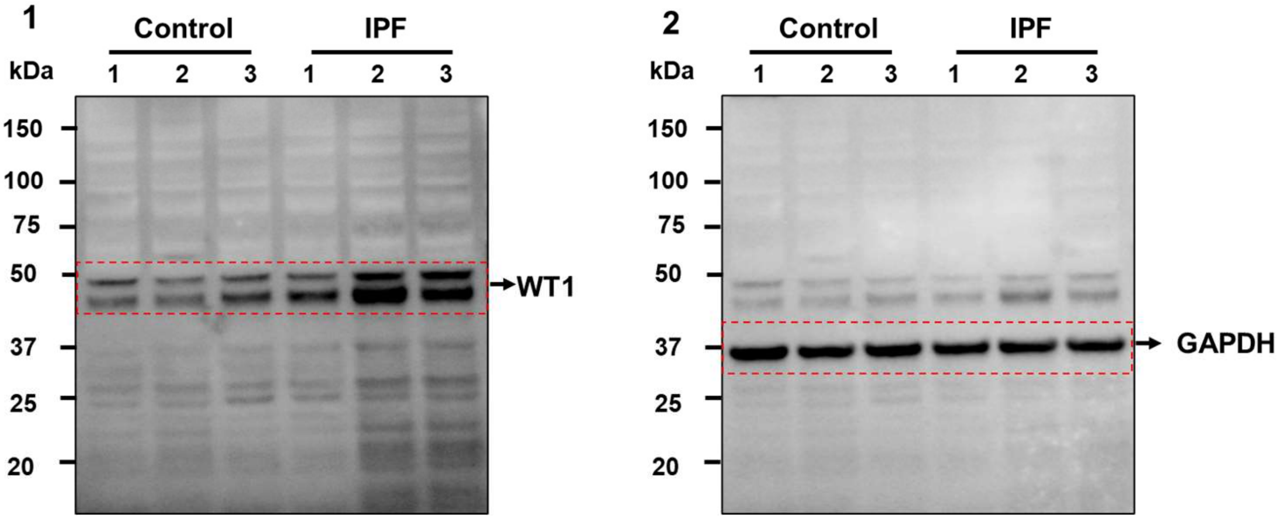

Full unedited blots for Figure 4C

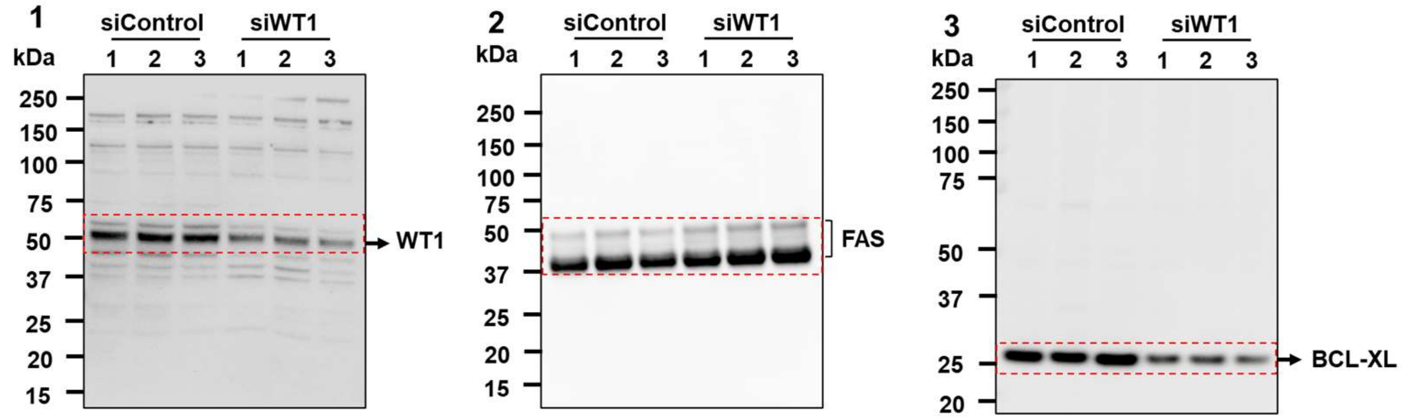

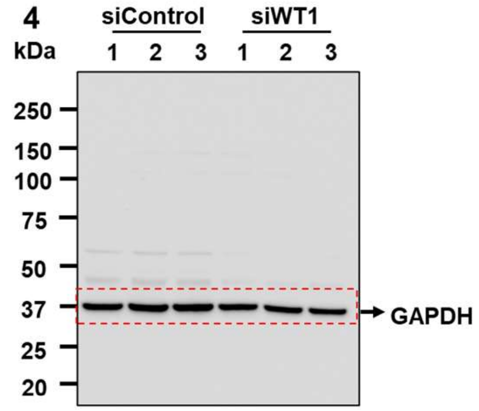

Full unedited blots for Figure 4E

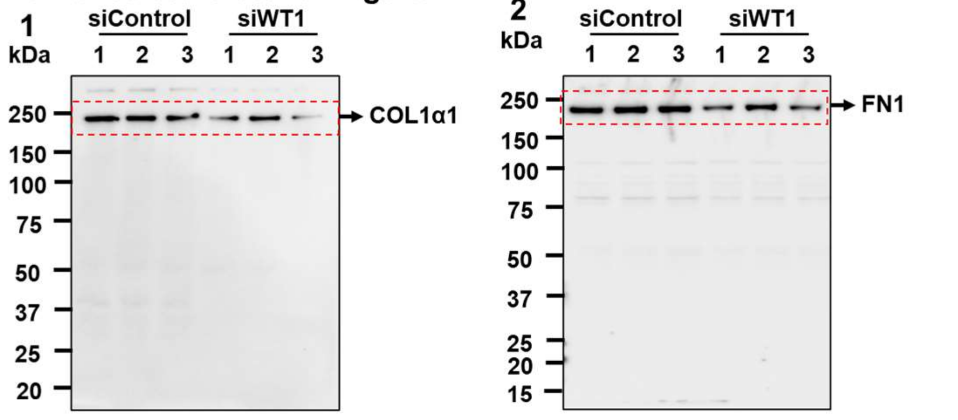

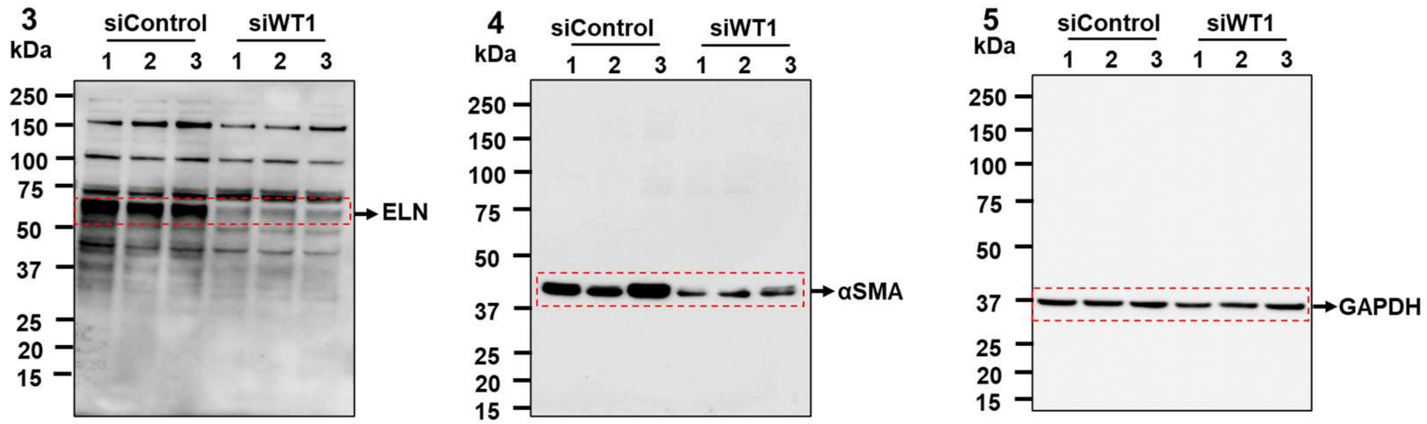

Full unedited blots for Figure 5D

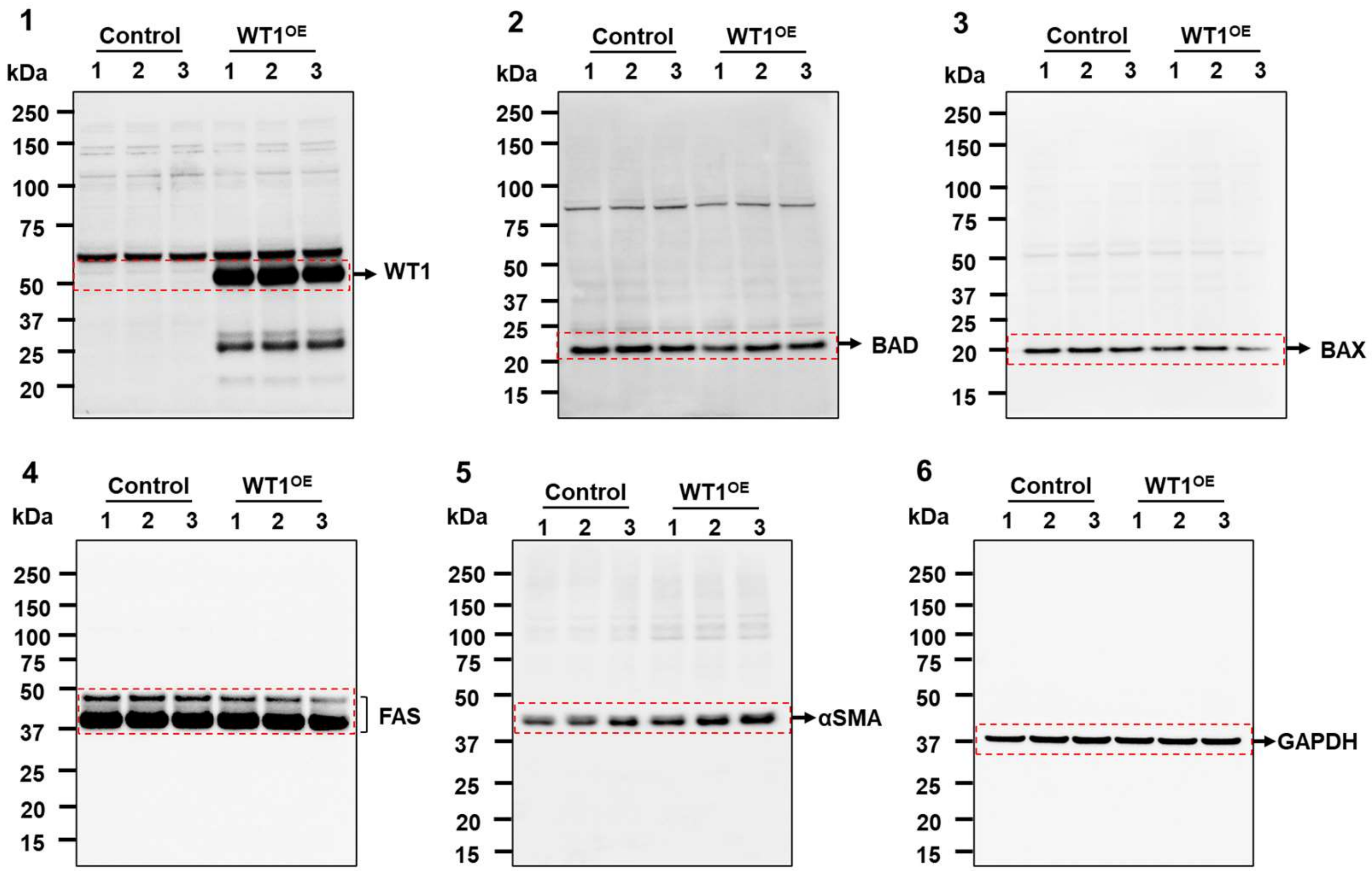

Full unedited blots for Figure 6B

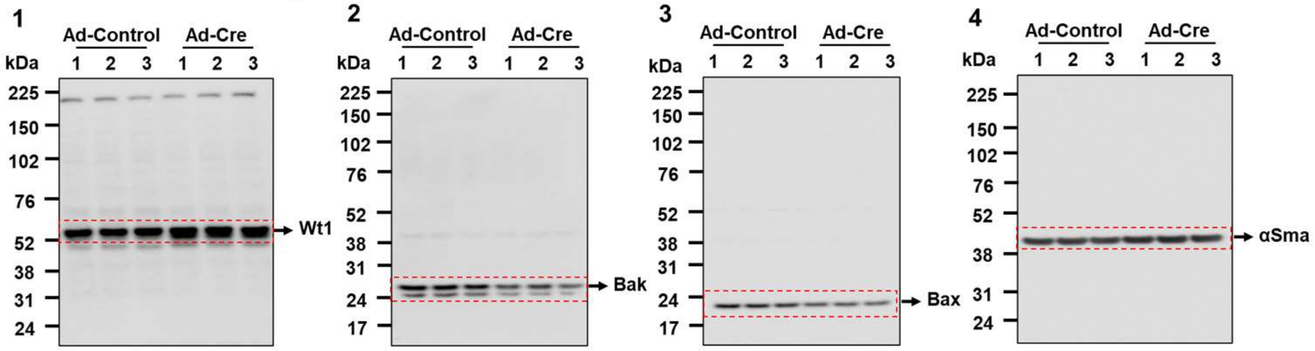

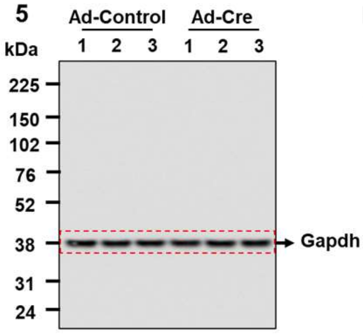

Full unedited blots for Figure 6E

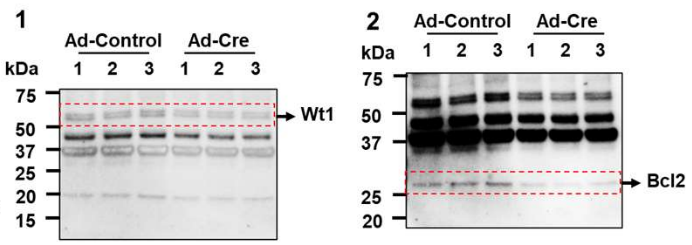

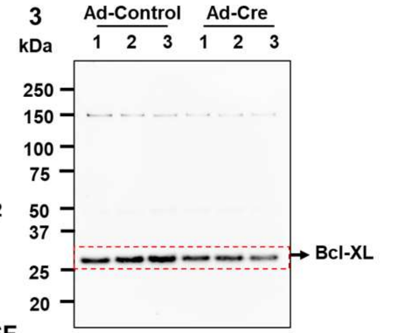

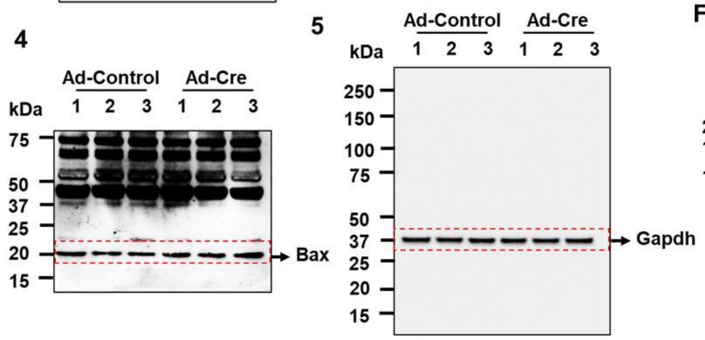

Full unedited blots for Figure 6F

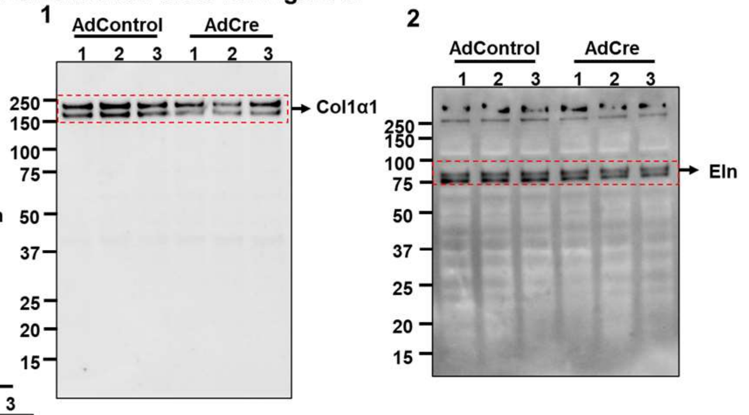

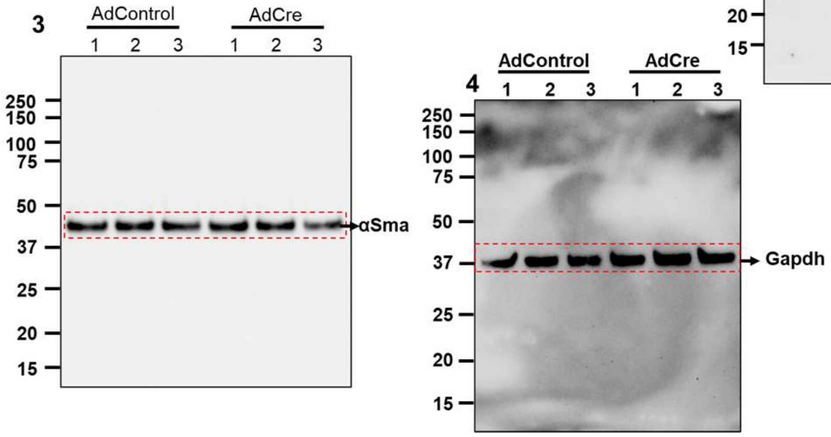

Full unedited blots for Figure 8C

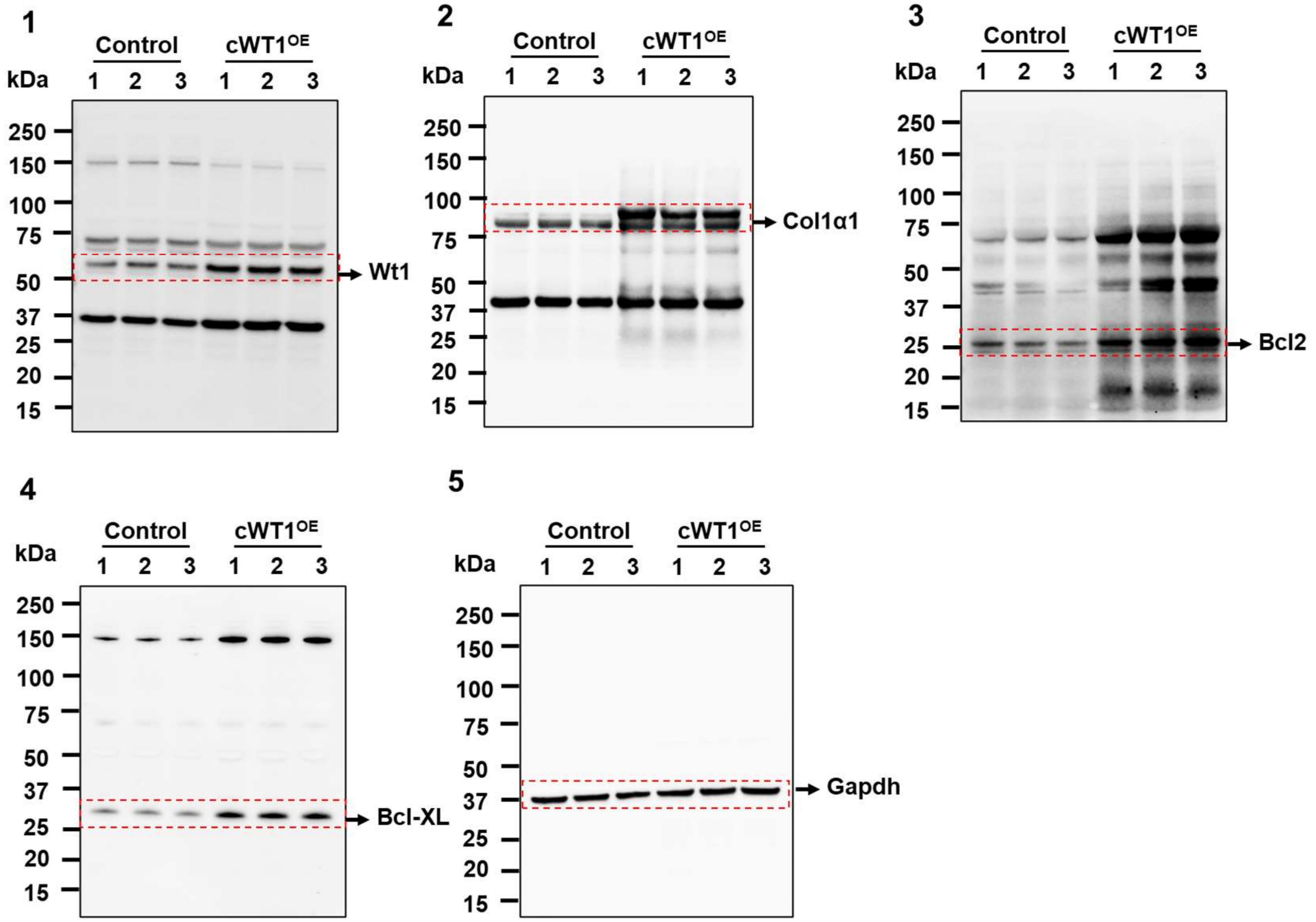

Full unedited blots for Supplementary Figure 14

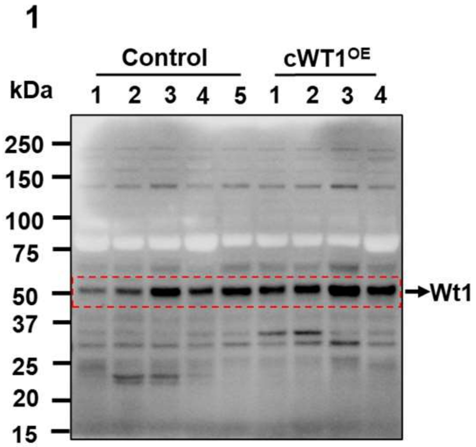

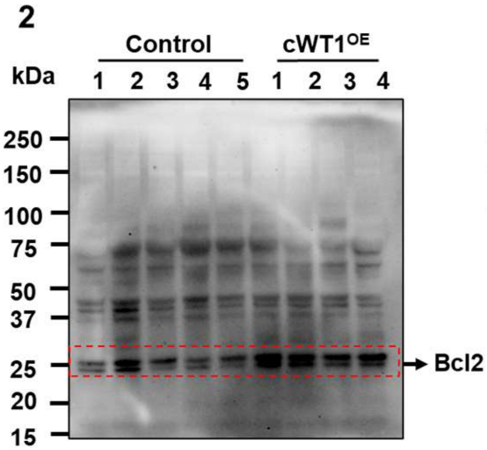

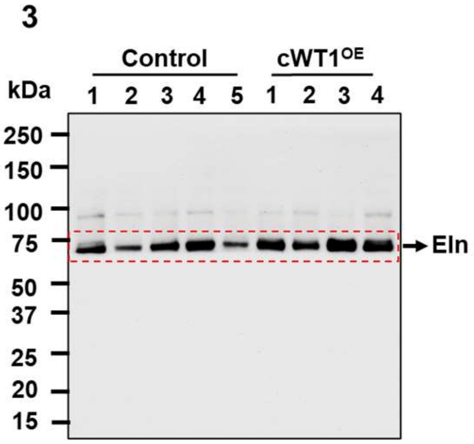

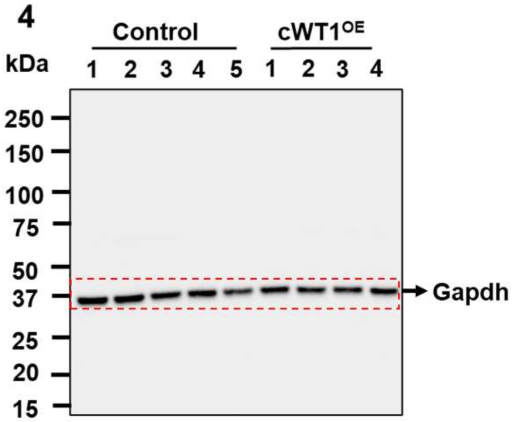

Supplement: Unedited blot and gel images [file jci-135-188819-s089.pdf]
